# Imaging Fluorescence Blinking of a Mitochondrial Localization Probe: Cellular Localization Probes Turned into Multifunctional Sensors

**DOI:** 10.1021/acs.jpcb.2c01271

**Published:** 2022-04-13

**Authors:** Zhixue Du, Joachim Piguet, Glib Baryshnikov, Johan Tornmalm, Baris Demirbay, Hans Ågren, Jerker Widengren

**Affiliations:** † Royal Institute of Technology (KTH), Experimental Biomolecular Physics, Department Applied Physics, Albanova Univ Center, 106 91 Stockholm, Sweden; ‡ Laboratory of Organic Electronics, Department of Science and Technology, Linköping University, SE-60174 Norrköping, Sweden; § Department of Physics and Astronomy, Uppsala University, P.O. Box 516, SE-751 20 Uppsala, Sweden

## Abstract

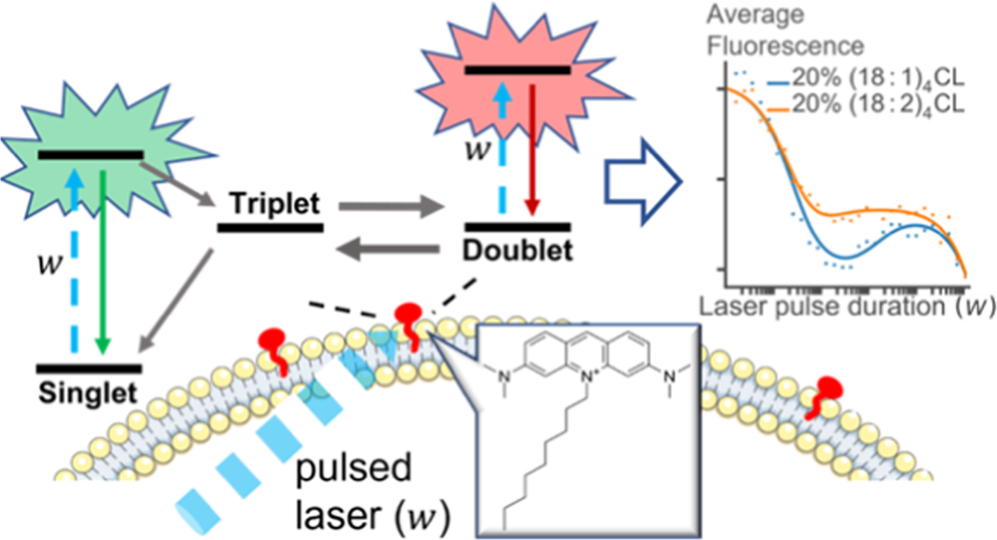

Mitochondrial membranes and their microenvironments directly influence and reflect cellular metabolic states but are difficult to probe on site in live cells. Here, we demonstrate a strategy, showing how the widely used mitochondrial membrane localization fluorophore 10-nonyl acridine orange (NAO) can be transformed into a multifunctional probe of membrane microenvironments by monitoring its blinking kinetics. By transient state (TRAST) studies of NAO in small unilamellar vesicles (SUVs), together with computational simulations, we found that NAO exhibits prominent reversible singlet–triplet state transitions and can act as a light-induced Lewis acid forming a red-emissive doublet radical. The resulting blinking kinetics are highly environment-sensitive, specifically reflecting local membrane oxygen concentrations, redox conditions, membrane charge, fluidity, and lipid compositions. Here, not only cardiolipin concentration but also the cardiolipin acyl chain composition was found to strongly influence the NAO blinking kinetics. The blinking kinetics also reflect hydroxyl ion-dependent transitions to and from the fluorophore doublet radical, closely coupled to the proton-transfer events in the membranes, local pH, and two- and three-dimensional buffering properties on and above the membranes. Following the SUV studies, we show by TRAST imaging that the fluorescence blinking properties of NAO can be imaged in live cells in a spatially resolved manner. Generally, the demonstrated blinking imaging strategy can transform existing fluorophore markers into multiparametric sensors reflecting conditions of large biological relevance, which are difficult to retrieve by other means. This opens additional possibilities for fundamental membrane studies in lipid vesicles and live cells.

## Introduction

Local conditions in cellular membranes modulate functions of membrane proteins and thereby largely determine cellular physiology on the whole. Proteins located in the inner mitochondrial membranes (IMMs) mediate a major part of the cellular metabolism and a multitude of other functions, including cellular apoptosis, production of reactive oxygen species (ROS), cellular signaling, and Ca^2+^ buffering. Here, also the IMMs themselves have a crucial modulating role and exhibit features and functions that set them apart from other cellular membranes.^1^ Particularly, the lipid composition of IMMs is different, in that they contain mainly phospholipids and a high content of the phospholipid cardiolipin (CL).

CL consists of a polar head group with two negative charges and four unsaturated acyl chains. CL has a critical role in the structure and stability of membranes involved in oxidative phosphorylation (OXPHOS) and is preferentially localized in membrane leaflets with high concave curvatures, in the cristae of the CL-rich IMMs, and in cell poles and dividing septa of bacterial membranes.^2^ At these membrane locations, CL interacts with many of the OXPHOS protein complexes^3^ and has been proposed to facilitate proton transfer between these proteins^4−6^ and along the membrane itself,^7^ thereby contributing to the overall OXPHOS efficiency.^8,9^ Alterations in CL are the main cause of mitochondrial defects, in turn underlying several pathologies, including neurodegeneration, cancer, diabetes, and cardiomyopathies.^10^

To understand the fundamental biology and pathophysiology of mitochondria, and the CL- and lipid-mediated effects involved, effective methods for the detection and quantification of CL and mitochondrial membranes are needed. Quantitative profiling of the lipidomics of the mitochondria and bacteria is possible with high-resolution mass spectrometry^11^ but requires sophisticated instrumentation and cannot be done on intact, live cells. Moreover, only a limited scope of local dynamical and environmental information about the membranes can be provided. Fluorescence-based analyses require less complicated instrumentation, are compatible with live cell studies, and can yield dynamical and environmental information with high sensitivity and specificity. However, such analyses rely on fluorescent probes specific for the mitochondrial membranes or CL, which at the same time preferentially should provide local environmental and dynamical information.

While there exist many mitochondrial localization probes, they rarely offer sensing capabilities of local, biologically relevant membrane conditions. The cationic dye 10-nonyl acridine orange (NAO) has since long been used for CL detection and mitochondria staining^9,12−14^ and is essentially the only commercially available dye for CL detection.^15^ NAO binds to anionic phospholipids, and with a specifically high affinity for CL, attributed to electrostatic interactions and hydrophobic interaction between adjacent NAO fluorophores when bound to the two phosphate residues of CL.^16^ NAO emits in the green (525 nm) when present in membranes as monomers but yields a characteristic red (640 nm) excimer fluorescence when associated with CL-rich membranes as π–π-stacked dimers.^9^ This CL quantification is however complicated by the fact that the red NAO emission requires a relatively narrow concentration range of NAO itself, around a NAO/CL mole ratio of 2:1, for reliable dimer formation.^15^ With lower NAO concentrations, formation of red-emitting dimers is limited, and with higher concentrations follows fluorescence self-quenching. The relatively high NAO concentrations required for dimer formation may also interfere with the membrane properties and with mitochondrial function. Apart from quantification of CL, with its limitations, and the mere localization of CL offered by NAO, little information about the local environment in the labeled membranes can be obtained.

To overcome these restrictions, we investigated the dark-state transition kinetics of NAO, as a readout of local environmental conditions in membranes of lipid vesicles and live cells, at concentrations where no NAO dimers are formed. Blinking behavior of fluorophores, as monitored by fluorescence correlation spectroscopy (FCS), has been shown to reveal their underlying triplet state,^17^ photoisomerization,^18,19^ and redox state^20^ transitions. These transitions can in turn sensitively reflect local environmental parameters around the fluorophores, such as oxygen concentration, viscosity, or redox conditions. Similarly, by analyzing the blinking of individual pH-sensitive fluorophores by FCS, as protons bind and dissociate from the fluorophores,^21^ it is possible to study local proton-exchange dynamics on biological membranes, how they can serve as proton collecting antennas, and thereby obtain a better understanding of their role in OXPHOS proton transport.^22,23^ However, FCS measurements rely on single-molecule detection conditions and on a high time resolution of the fluorescence detection. The use of FCS as a blinking readout is thus limited to bright, photostable fluorophores and to samples where single-molecule signal-to-background conditions can be achieved. By transient state (TRAST) monitoring,^24,25^ these limitations of FCS can be overcome. TRAST determines the blinking kinetics of fluorescent molecules from how their time-averaged fluorescence intensity varies with the modulation of the laser excitation intensity. Like FCS, it combines a high detection sensitivity offered by the fluorescence signal, with a high environmental sensitivity, offered by the long-lived dark transient states. However, TRAST does not rely on single-molecule detection conditions and allows studies based on dim, autofluorescent compounds,^26,27^ and also in living cells.^28^ On the other side, only photoinduced dark-state transitions can be analyzed by TRAST, which excludes the use of most pH-sensitive fluorophores for proton-exchange studies under relevant biological conditions.

In this work, we studied NAO by TRAST, implemented into a standard wide-field microscope. From the TRAST measurements, supported by computational simulations, we found that NAO exhibits prominent singlet–triplet state transitions and acts as a light-induced Lewis acid. Following triplet state formation, NAO can form weakly bonded complexes with hydroxyl ions, in turn leading to formation of charge-transfer complexes ^**3**^[NAO^•^ OH^•^], which upon dissociation leads to the formation of a red-emissive doublet radical. The reversible transitions to and from the triplet and doublet radical states of NAO, clearly observed by TRAST imaging, were found to reflect local oxygen concentrations, redox conditions, presence of radicals, the charge of membrane lipid head groups, and the molar ratio of CL in the membranes. Notably, they could also reflect pH and buffer capacity, which are normally not captured by photoinduced dark-state transitions analyzed in TRAST. These parameters are all important for OXPHOS in IMMs and bacterial membranes. This study suggests an extended use of NAO, not only as a localization probe but also as a multiparametric sensing probe for cellular and membrane studies. More generally, with the presented strategy, imaging fluorescence blinking in cells and membranes, a large group of already existing fluorophore localization probes can be turned into sensors of local membrane environments.

## Methods

### TRAST Spectroscopy

In TRAST measurements, fluorophore blinking kinetics are determined by recording the average fluorescence intensity from an ensemble of fluorophores subject to modulated excitation. With the excitation modulation systematically varied on the time scales of the fluorophore dark-state kinetics, rapid blinking kinetics can be quantified without the need for time-resolved detection.^24,25^ This enables wide-field cellular imaging of μs blinking kinetics, using a regular camera and exposure times of seconds.

To calculate the recorded fluorescence intensity in the TRAST experiments, we used the photophysical model for NAO with two states emissive upon excitation, the singlet state ^1^NAO^+^ and the doublet radical state ^2^NAO^•^, and with the other states in the model (the triplet state, ^3^NAO^+^, and the bleached state, B) nonluminescent, see the Results section. For a homogeneous solution sample, and from the rate equations of a NAO fluorophore subject to a rectangular excitation pulse starting at *t* = 0 (Supporting Information, Section S1 and eqs S1–S6), the fluorescence signal recorded in our experimental setup can be described by
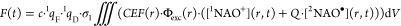
1Here, [^1^NAO^+^] and [^2^NAO^•^] denote the probabilities that each of these emissive states (in either their ground or excited states) is populated in the fluorophores and *Q* = (^2^*q*_F_·^2^*q*_D_·σ_2_)/(^1^*q*_F_·^1^*q*_D_·σ_1_) is the relative brightness of [^2^NAO^•^] compared to [^1^NAO^+^], where ^1^*q*_D_ and ^2^*q*_D_ denote the overall detection quantum yield of the emission from the excited singlet state, _1_^1^NAO^+^*, and the excited doublet radical state, _1_^2^NAO^•^*, respectively, and ^1^*q*_F_ and ^2^*q*_F_ are the fluorescence quantum yields of these states. *CEF*(*r̅*) is the collection efficiency function of the detection system and *c* is the fluorophore concentration.

At the onset of excitation, *F*(*t*) will show characteristic relaxation on a μs to ms time scale, reflecting changes in the population of the emissive state(s) (see the Supporting Information, Section S1 and eqs S1–S6). Similar relaxations can also be observed in the time-averaged fluorescence signal resulting from a rectangular excitation pulse of duration *w*
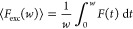
2as *w* is increased from the μs to the ms time range. Analyzing how ⟨*F*_exc_(*w*)⟩ varies with *w* then allows the population kinetics of long-lived photoinduced states of the fluorophore to be determined, which is the general basis for TRAST monitoring.^25^

To obtain sufficient photon counts, even for short *w*, we collected the total signal resulting from an excitation pulse train of *N* identical pulse repetitions. *N* is adjusted to maintain a constant laser illumination time, *t*_ill_ = *N*·*w* = 10 ms, for all *w*. A so-called TRAST curve is then produced by calculating the time-averaged fluorescence signal during excitation for each pulse train, normalized for a given pulse duration, *w*_0_
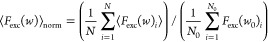
3The pulse duration used for normalization, *w*_0_, is chosen to be short enough (typically sub-μs) not to lead to any noticeable build-up of dark transient states, yet longer than the antibunching rise time of *F*(*t*) upon onset of excitation, which typically is in the nanosecond time range.^29^

In the above expression, ⟨*F*_exc_(*w*)_*i*_⟩ represents the total signal collected from the *i*th pulse in the pulse train, as defined in eq 2. By using a low excitation duty cycle, here η = 0.01, fluorophores are allowed to fully recover back to S_0_ before the onset of the next pulse, making all pulses in a given pulse train identical. The summations in eq 3 are then no longer required and the expression simplifies further. Of note, in the normalization step of eq 3, several constants used to calculate *F*(*t*) in eq 1 cancel out. The final expression for ⟨*F*_exc_(*w*)⟩_norm_ therefore becomes independent of *c* and the absolute *q*_D_ and *q*_F_ values for the two emissive species.

A complete TRAST experiment consisted of a stack of 30 fluorescence images. Each image represents the total fluorescence signal from an entire excitation pulse train, captured using a camera exposure time of *t*_exp_ = *t*_ill_/η = 1 s. Pulse durations, *w*, were distributed logarithmically between 100 ns and 10 ms and were measured in a randomized order to avoid bias due to time effects. An additional 10 reference frames, all using 100 ns pulse duration to avoid dark-state build-up, were inserted at regular intervals between the 30 main images to track any permanent bleaching of the sample.

Electronic state model for NAO, see the Supporting Information, Section S1.

Spatial distribution of excitation rates, calculation of average rates, see the Supporting Information, Section S2.

Preparation of lipid vesicles, see the Supporting Information, Section S3.

Cell preparation, see the Supporting Information, Section S4.

### Experimental Setup for TRAST Measurements

**Figure 1:**
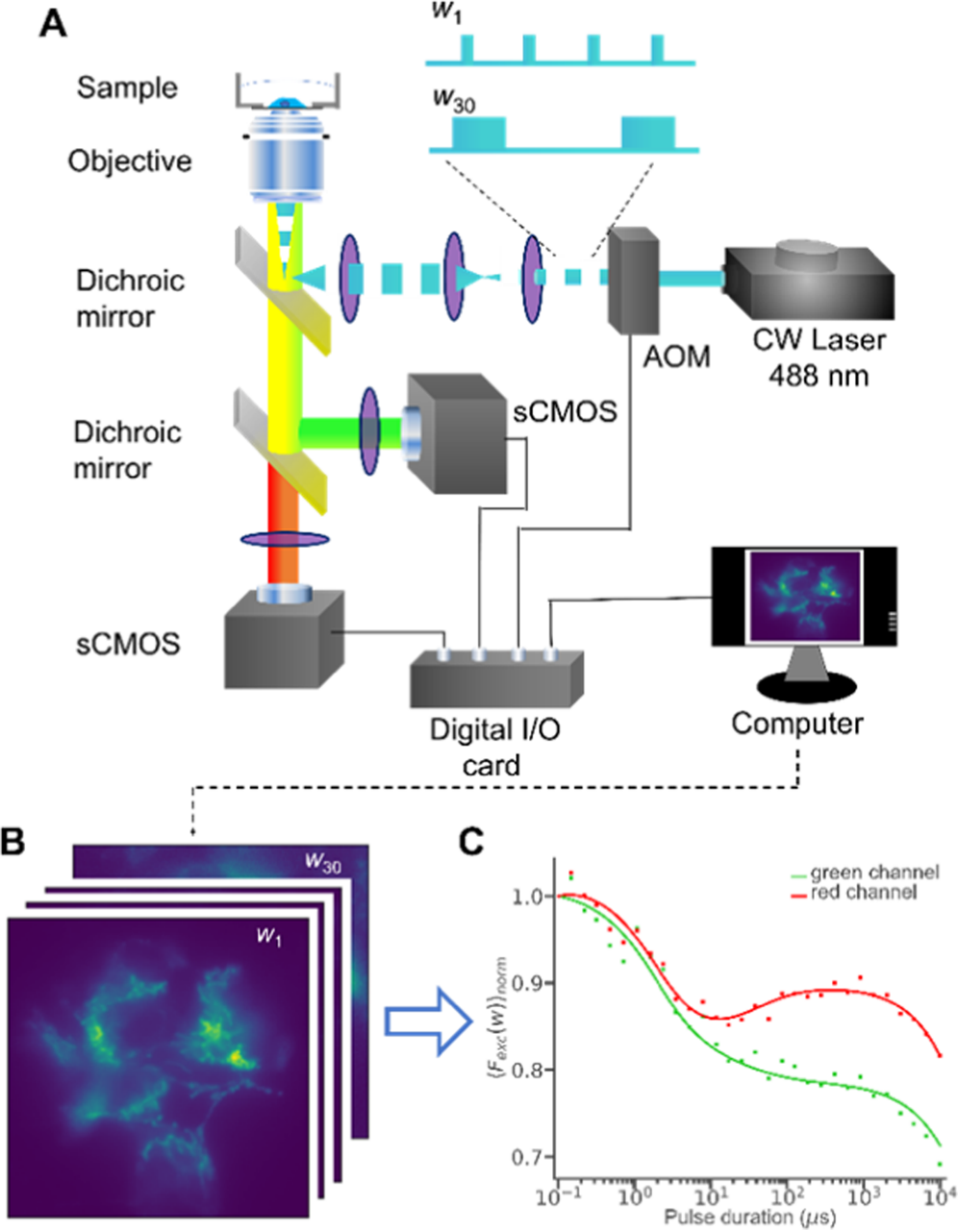
Schematic representation of the wide-field TRAST experiments. (A) An excitation laser is modulated by an acousto-optic modulator (AOM) to produce low-duty cycle, rectangular excitation pulse trains with different pulse durations, *w*. The time-averaged fluorescence signal from the sample is detected by two scientific complementary metal oxide semiconductor (sCMOS) cameras in two different emission wavelength ranges (green and red). Wide-field images (B) are acquired using excitation pulse trains with different pulse durations (only images from the red channel shown). TRAST curves can then be obtained (C) showing how the normalized, detected time-averaged fluorescence intensity, ⟨*F*_exc_(*w*)⟩_norm_, recorded in a specific region of interest (ROI) of the sample varies with *w*. These curves reflect how the fluorescence intensity changes after the onset of an excitation pulse and allow the determination of the dark-state transitions within the fluorophores of the sample (see the Methods section for details).

TRAST measurements were carried out on a home-built TRAST setup (Figure 1A), as previously described,^30^ see the Supporting Information, Section S5.

TRAST data analysis was based on a software implemented in Matlab, as previously described,^30^ see the Supporting Information, Section S6.

TRAST images of cells, see the Supporting Information, Section S7.

Fluorescence lifetime measurements, see the Supporting Information, Section S8.

Spectroscopic Measurements, see the Supporting Information, Section S9.

Computational details for the fluorophore simulations, see the Supporting Information, Section S10.

## Results

### TRAST Measurements of the NAO-Labeled POPC Vesicle—Formation of a Red-Emissive Species and Effects of Oxygenation, Excitation Intensity, and pH

Small unilamellar vesicles (SUVs) of POPC labeled with NAO were prepared in dye concentrations where the dye does not form dimers, as described (see the Methods section). Although NAO prefers binding to anionic phospholipids, and with a specifically high affinity for CL, NAO can also bind to other lipid membranes via the nonyl chain of NAO.^9^ Compared to NAO in Dulbecco’s phosphate-buffered saline (DPBS) buffer, the fluorescence intensity of NAO significantly increases when vesicles are present (Figure S1). The SUVs were subject to TRAST measurements with 488 nm excitation and with the fluorescence recorded in two separate spectral channels, centered in the green (530 nm) and red (670 nm) spectral range, using two sCMOS cameras (Figure 1).

**Figure 2:**
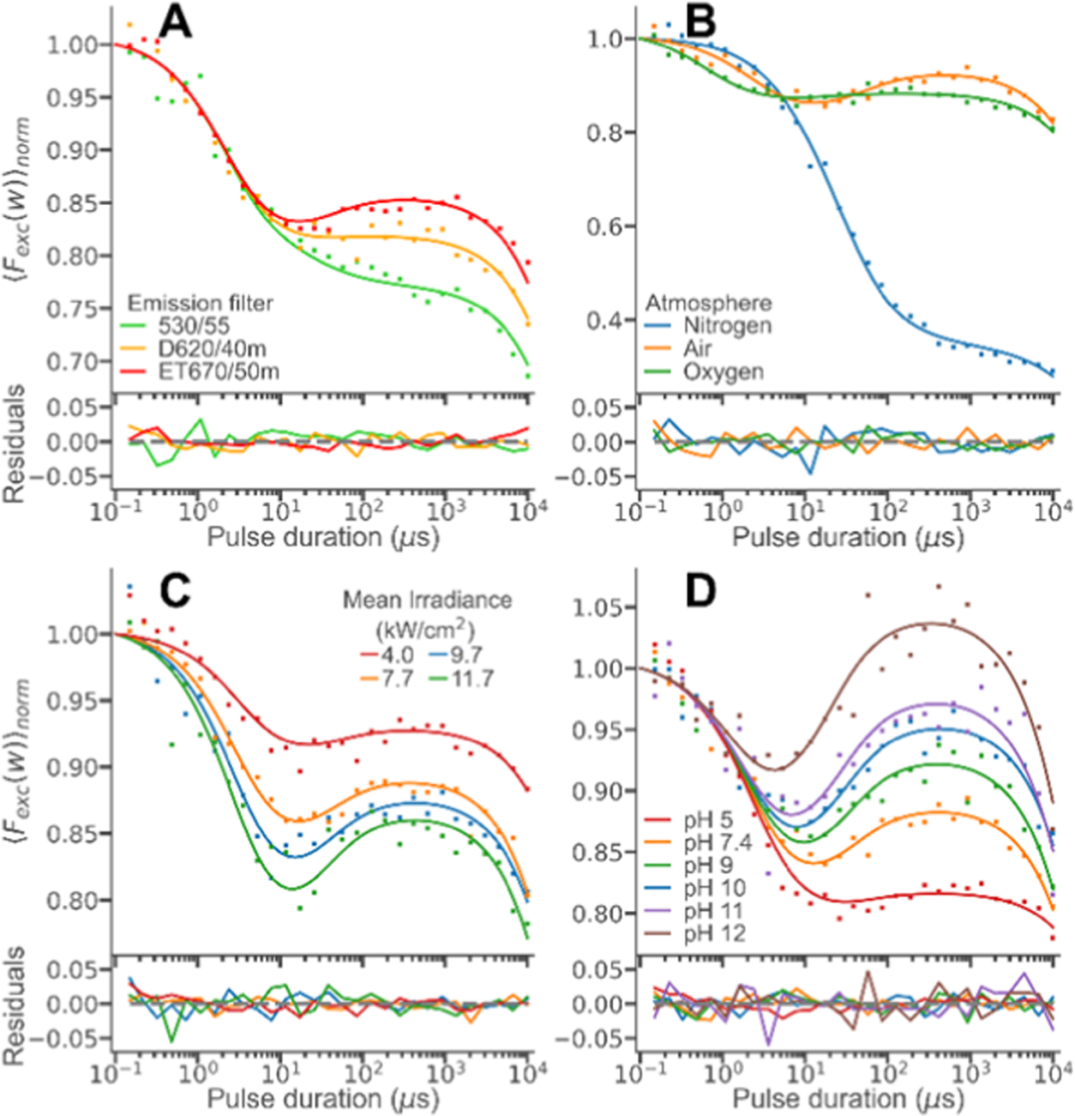
Experimental TRAST curves recorded from POPC SUVs labeled with NAO in DPBS buffer (pH 7.4, air atmosphere, *I*_exc_ 11.7 kW/cm^2^, if not stated otherwise). The TRAST curves were fitted globally using the photophysical model of Figure 3B (see the main text for details), and the fitting residuals are plotted below the curves. (A) TRAST curves recorded in different fluorescence emission wavelength ranges. (B) TRAST curves recorded under different atmospheres/[O_2_] (air, pure nitrogen, and pure oxygen). (C) TRAST curves recorded under different excitation irradiances, *I*_exc_. (D) TRAST curves recorded from the SUVs, with the DPBS buffer set to different pH.

In the TRAST measurements, the average fluorescence intensity from the SUVs was recorded upon excitation with different, low-duty cycle, rectangular excitation pulse trains.^25−27,30^ The recorded intensities, ⟨*F*_exc_(*w*)⟩, were then plotted as a function of the duration, *w*, of the excitation pulses, in so-called TRAST curves (see the Methods section for details). Representative TRAST curves obtained from POPC SUVs labeled with NAO and recorded in different emission wavelength ranges are shown in Figure 2A. While all curves decay in the microsecond time range, representing fluorescence decay due to triplet state (T_1_) build-up, the red curve shows an increase in the time range of 10–100 ms, not seen in the green TRAST curve. This indicates that following the onset of excitation, a red-emissive state is formed in NAO on this time scale. To elucidate the identity and formation mechanisms of this red-emissive state, we hereinafter focused our TRAST experiments on the red emission range of the NAO fluorescence. That the observed decay in the TRAST curves of Figure 2A indeed can be attributed to triplet state build-up was further supported by TRAST curves recorded at different oxygen concentrations [O_2_], on samples in pure oxygen, air, or nitrogen atmospheres (Figure 2B). Oxygen is a potent triplet state quencher, and the recorded TRAST curves are well in agreement with the higher triplet state population and slower relaxation previously found in FCS^17^ and TRAST^24,25^ measurements for organic fluorophores at low [O_2_]. We then recorded TRAST curves with different excitation intensities, *I*_exc_, onto the sample (Figure 2C), confirming that both the triplet state of NAO and its red-emissive state are photoinduced and that both populations increase with higher *I*_exc_. Moreover, in the TRAST curves recorded at high [O_2_] (Figure 2B), when the triplet state population is suppressed, the increase due to the red-emissive state is also very small, suggesting that this state is primarily formed from the T_1_ state. Further, variation of pH showed that the formation of the red-emissive state is highly pH-dependent and that its formation is favored under high pH (Figure 2D). Given the pH and *I*_exc_ dependence found in the TRAST experiments, we performed fluorescence lifetime measurements on the same NAO samples under different pH and *I*_exc_. The fluorescence decay could be fitted as a biexponential decay, with two lifetimes of 1.6 and 4.6 ns, where the relative fraction of the longer lifetime increased with both *I*_exc_ and pH (Figure S2). This gives further evidence for an excitation-induced, red-emissive species, promoted at higher pH, and with an excited state lifetime of 4.6 ns. From spectrofluorometer measurements, this red-emissive species is barely noticeable, as a slight redshift of the emission spectrum at higher pH (Figure S3). However, since the red-emissive species is excitation-induced and since the excitation intensity used in a regular spectrofluorometer is low, as compared to the *I*_exc_ used in the TRAST and fluorescence lifetime measurements (see the Methods section), such a minor contribution of a red-shifted emission is to be expected.

Similar to the TRAST curves recorded from the NAO-labeled SUVs in this study, a pH- and excitation-dependent rise process has previously been found in TRAST curves recorded from free tryptophan (Trp) in low-pH buffer solutions^26^ and also in FCS studies of fluorescent photoacids under single-molecule detection conditions.^31^ This rise process can be attributed to a higher p*K*_a_ of the ground singlet state (S_0_) than the excited singlet state (S_1_) of the fluorescent molecules studied. In TRAST curves recorded from Trp at a low pH,^26^ lower than the p*K*_a_ of the S_0_ state, but still higher than that of the S_1_ state, Trp can upon excitation be deprotonated. Since the deprotonated form of Trp shows a stronger fluorescence than the protonated form, the onset of excitation drives Trp into a more fluorescent form, and a rise in the overall fluorescence can be observed in the TRAST curves with increasing *w*. We investigated the dye Acridine Orange (AO), which is very similar to NAO except for the *n*-nonyl group and which is known to be a stronger base in the S_1_ state (p*K*_a_ = 13.3) than in the T_1_ and S_0_ states (p*K*_a_ = 10.3 and 10.2, respectively).^32^ TRAST curves recorded from AO at 488 nm excitation confirm that AO can act as a photobase and that excitation at a pH between 10 and 13 can drive AO into a protonated, more fluorescent state (Figure S4). In contrast to NAO, however, no rise was found in the TRAST curves recorded from AO at physiological pH (∼7.4), indicating that its protonation state was not influenced by excitation at this pH. Moreover, the site of proton exchange in AO is the intracyclic nitrogen atom,^32^ which in NAO is substituted by the *n*-nonyl group. The nitrogen is thus not available as a protonatable group in NAO. Together with the finding that pH titration of NAO does not show any p*K*a in the range of pH 2.2 to 10^33^ further indicates that the rises in the TRAST curves recorded from AO and NAO have different origins. The absence of evidence for NAO acting as a photobase or photoacid, based on shifts in its p*K*_a_ upon excitation, led us to consider if NAO can act as an electron pair acceptor, a so-called Lewis acid. For certain organic molecules,^34^ Lewis acidity can be enhanced by photoexcitation. For NAO, the correlation between triplet state and red-emissive state formation suggests an increased capability to accept an electron pair after excitation and intersystem crossing into its triplet state. The pH dependence of the red-emissive species indicates that the formation of this species is promoted by OH^–^ ions binding to cationic NAO in its triplet state, thereby acting as the corresponding Lewis base.

### Photophysical Model for NAO

To add evidence and further investigate the prerequisites for NAO to act as a light-induced Lewis acid, computational modeling was performed (see the Supporting Information, Section S11 and Figure S5, for details). The computational calculations support the view that upon onset of excitation in the TRAST measurements and following ^1^NAO^+^ → ^3^NAO^+^ intersystem crossing, side-track formation of a charge-transfer complex between ^3^NAO^+^ and hydroxyl ions (OH^–^), ^**3**^[NAO^•^ OH^•^], can take place. Dissociation of this complex can then subsequently lead to the generation of doublet radicals, ^2^NAO^•^. This ^2^NAO^•^ species is responsible for a red-shifted emission, with a longer fluorescence lifetime component, as observed experimentally (Figure S2) and as predicted from rate parameter calculations.^35^ The computational calculations are also consistent with the observed increase in this component with higher *I*_exc_ (Figure 2C).

**Figure 3:**
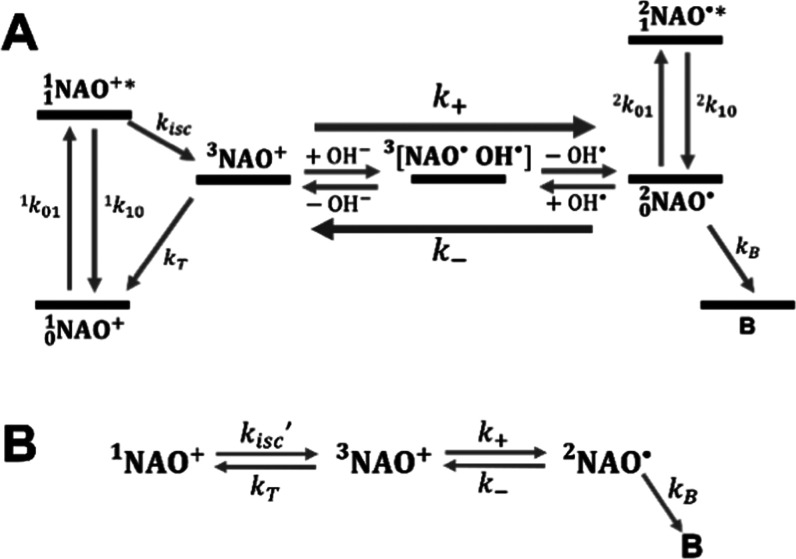
(A) Photophysical model for NAO. Excitation takes place from the singlet ground state, _0_^1^NAO^+^, to the first excited singlet state, _1_^1^NAO^+^*. Excitation can result in dark-state formation, via intersystem crossing to a triplet state, (^3^NAO^+^), which in turn can relax back to _0_^1^NAO^+^ or form a ^**3**^[NAO^•^ OH^•^] complex with hydroxyl ions in the solution. Dissociation of this complex can then subsequently lead to the generation of a doublet radical, _0_^2^NAO^•^, and a hydroxyl radical, OH^•^. _0_^2^NAO^•^ can be excited into an emissive excited state, _1_^2^NAO^•^*, and may also react with a hydroxyl radical into a ^**3**^[NAO^•^ OH^•^] complex, which upon dissociation of the hydroxyl ion can be transformed back to ^3^NAO^+^. At the NAO concentrations used in this study, dimerization can be neglected^16^ and was therefore not included in the model nor were exciplex formation and triplet–triplet annihilation processes. At the excitation intensities used in the experiments, excitations to higher singlet or doublet excited states can be neglected and were also not included in the model. Rate parameters: excitation rate of _0_^1^NAO^+^ (^1^*k*_01_ = σ_1_·Φ_exc_, where σ_1_ is the excitation cross section of _0_^1^NAO^+^ and Φ_exc_ is the excitation photon flux), combined fluorescence and nonradiative decay rate of _1_^1^NAO^+^* (^1^*k*_10_), intersystem crossing rate (*k*_isc_), triplet relaxation rate (k_T_), compound formation and recovery rates of the doublet radical from the triplet state (*k*_+_ and *k*_–_), excitation rate of _0_^2^NAO^•^ (^2^*k*_01_ = σ_2_·Φ_*exc*_, where σ_2_ is the excitation cross section of _0_^2^NAO^•^), and combined fluorescence and nonradiative decay rate of _1_^2^NAO^•^* (^2^*k*_10_). Finally, additional degradation pathways of the doublet radicals into a degraded, dark state, B, were included as a rate, *k*_B_. (B) Photophysical model for the TRAST analyses. Both the ^3^NAO^+^ and the ^**3**^[NAO^•^ OH^•^] complex state in Figure 3A are nonfluorescent, and transitions between these states cannot be distinguished in the TRAST measurements. In the TRAST analyses, the transitions between the triplet and the doublet radical states were therefore modeled as compound rates, *k*_+_ and *k*_–_, with the ^**3**^[NAO^•^ OH^•^] complex not included as a separate state in the photokinetic model. Additionally, given lifetimes in the nanosecond range for the excited emissive states _1_^1^NAO^+^* and _1_^2^NAO^•^*, the equilibrations with their corresponding ground states upon onset of excitation are both much faster than the other state transitions in the model. In the TRAST measurements, monitoring these other transitions, we could therefore restrict ourselves in the model to include only one joint, equilibrated state for the singlet and one for the doublet state, denoted ^1^NAO^+^ and ^2^NAO^•^, respectively. Consequentially, we then also replaced *k*_isc_ with the effective intersystem crossing rate, *k*_isc_′ = *k*_isc_[σ_1_·Φ_exc_/(σ_1_·Φ_exc_ + ^1^*k*_10_)]. Finally, the doublet state relaxation in the TRAST curves typically occurs at an order of magnitude longer time scale than the singlet–triplet state relaxation. While relaxation of ^2^NAO^•^ may occur both in the singlet and triplet states, it is difficult to kinetically distinguish these transitions from each other. We therefore only included one of the rates (*k*_–_) in the model, the one from ^2^NAO^•^ to the triplet state ^3^NAO^+^. See the Supporting Information, Section S1, for the corresponding equations of this model.

Thus, based on the experimental data (Figure 2A–D), showing a population build-up of NAO^+^ in its triplet state upon onset of excitation, a pH- and *I*_exc_-dependent formation of a red-emissive species, and further supported by the computational simulations, we could set up a seven-state photophysical model for NAO^+^ (Figure 3A). For numerical fitting of the experimental TRAST curves, this model could be simplified to a four-state model (Figure 3B). With both the singlet state, ^1^NAO^+^, and the radical doublet state, ^2^NAO^•^, set as emissive states in the model, the detected fluorescence at time, *t*, after the onset of a rectangular excitation pulse, *F*(*t*), can be calculated by (eq 1 in the Methods section and eq S6) with *F*(*t*) linearly dependent on the population probability of both these states. This also holds for the normalized, detected time-averaged fluorescence intensity, ⟨*F*_exc_(*w*)⟩_norm_, as well as for the TRAST curves, showing how ⟨*F*_exc_(*w*)⟩_norm_ varies with the duration, *w*, of the excitation pulse (eq 3). Fitting of the photophysical rate parameters could then be performed by simulating theoretical TRAST curves based on the photophysical model of Figure 3B and by comparing the simulated curves to the experimental data (see the Methods section for details). In the fits, we used a reported excitation cross section (at 497 nm)^36^ for ^1^NAO^+^ of σ_1_ = 25.3·10^–17^ cm^2^ and its absorption spectrum to calculate the excitation cross section (at 488 nm) for ^1^NAO^+^ of σ_1_ = 20.8 × 10^–17^ cm^2^ and then to calculate the singlet state excitation rate (^1^*k*_01_ = σ·Φ_exc_) with the local photon flux calculated as Φ_exc_ = *I*_exc_λ/(*hc*), where *hc*/λ is the photon energy. The same excitation rate could also be assumed for ^2^NAO^•^, with the difference in excitation rates accounted for within *Q* = (^2^*q*_F_·^2^*q*_D_·σ_2_)/(^1^*q*_F_·^1^*q*_D_·σ_1_), representing the relative detected brightness of ^2^NAO^•^, compared to that of ^1^NAO^+^ (see eq 1). In the fits, the following parameters were thus fitted: *k*_isc_, *k*_T_, *k*_+_, *k*_–_, *k*_B_, and *Q*.

To evaluate the photophysical model (Figure 3B), we first fitted the experimental TRAST curves measured in the different emission bands and under different oxygen concentrations, excitation intensities, and pH (Figure 2). Several of the fitted parameter values obtained were then used and kept fixed in the fitting of the subsequent experimental TRAST curves. Further details of this fitting procedure are given in the Supporting Information, Section S12 and Figures S6,S7. Taken together, fitting the TRAST curves in Figure 2A–D confirms that the photophysical model of Figure 3B can adequately take the emission wavelength, excitation intensity, pH, and oxygenation dependence into account and that it is consistent with the two emissive states of NAO. The resulting fitted parameter values were 0.40, 16.2, 0.53, 0.077, and 0.042 μs^–1^ for *Q*, *k*_isc_, *k*_T_, *k*_+_, and *k*_–_, respectively (summarized in the Supporting Information, Table S1). These values were then used as fixed values in the fitting of subsequent experimental TRAST curves. From the fitting, the obtained *k*_B_ values were found not to vary significantly, they were at least two orders of magnitude lower than the other fitted rate parameters and did not significantly influence these parameters. We therefore do not further discuss the fitted *k*_B_ values.

To further verify the photophysical model of Figure 3A, we also investigated if spin labels (doxyl, added to SUVs labeled with NAO) and variations in the local redox environment (adding sodium ascorbate (NaAc) or hydrogen peroxide (H_2_O_2_) into the SUV solution) could influence the transition between the triplet and the doublet state of NAO. In FCS and TRAST measurements, addition of paramagnetic spin labels can be clearly observed to enhance the transitions between fluorophore singlet and triplet states. This can also be used for low-frequency collisional interaction or compartmentalization studies in biological membranes and cells.^30,37^ Likewise, addition of antioxidants can strongly influence dark-state transitions of many fluorophores, promoting recovery of photo-oxidized fluorophores and thus enhancing fluorescence emission but also enhancing photoreduction.^20^ The experimental TRAST data show similar prominent effects on the NAO dark-state transitions (see Supporting Information, Section S13 for details). This suggests that dark-state transitions in NAO can also be used to monitor molecular interaction in membranes and variations in local redox environments and further confirms the photophysical model of Figure 3.

### Electrostatic and Buffer Effects

**Figure 4:**
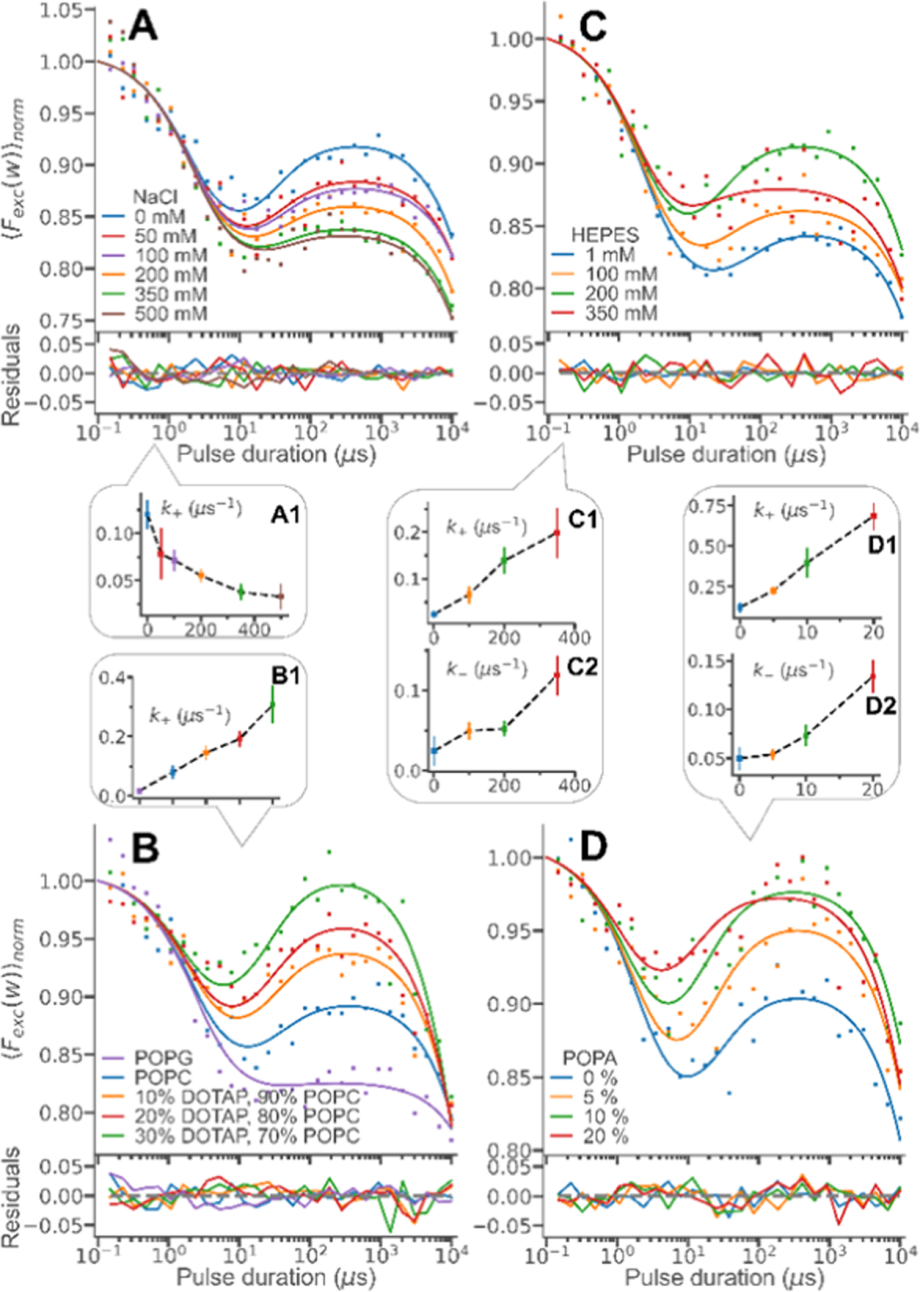
Experimental TRAST curves from SUVs labeled with NAO (DPBS buffer at pH 7.4, air atmosphere, measured at 11.7 kW/cm^2^), fitted globally using the photophysical model (Figure 3B), as described in the main text. (A) TRAST curves recorded from samples with different salt concentrations. Inset (A1): individually fitted *k*_+_ rates. (B) TRAST curves recorded from SUVs with different lipid compositions. The individually fitted *k*_+_ rates are shown in the inset (B1). (C) TRAST curves recorded from SUV samples with different HEPES buffer concentrations but with the same salt concentration (250 mM NaCl). Insets show the individually fitted rates *k*_+_ (C1) and *k*_–_ (C2). (D) TRAST curves recorded from SUVs with different molar fractions of POPA. Insets D1 and D2 show the fitted rates *k*_+_ and *k*_–_, respectively.

As suggested in the photophysical model (Figure 3A), formation of NAO doublet state radicals occurs via a charge-transfer complex ^3^[NAO^•^ OH^•^], preceded by the binding of hydroxyl ions to ^3^NAO^+^. As our computational simulations show, this binding is promoted by electrostatic attraction between cationic NAO and anionic hydroxyl ions. By increasing salt concentrations, we could clearly observe the shielding of this electrostatic attraction in our TRAST measurements, as a markedly lowered formation of NAO doublet state radicals (Figure 4A). To fit the parameters to the curves, *k*_T_, *k*_isc_*k*_–_, and *Q* were fixed to values as determined above (Supporting Information, Table S1), and *k*_+_ was fitted individually to each of the curves. The fitted curves were well in agreement with the measured TRAST curves and with *k*_+_ monotonously decreasing with increasing salt concentrations (Inset A1, Figure 4A). Next, we investigated the influence on the electronic state transitions of NAO from the charge of lipid head groups, located in the same vesicle membranes. SUVs with NAO were prepared, with POPG (negatively charged head group), POPC (zwitterionic head group), and POPC together with different molar fractions of DOTAP (positively charged head groups). The recorded TRAST curves from these vesicle samples displayed a significant increase in NAO doublet radical state formation with increasing amounts of positively charged lipid head groups in the membranes (Figure 4B). This is consistent with a stronger electrostatic attraction, and thereby a higher local concentration, of hydroxyl ions at the membranes, which promotes the *k*_+_ rate. The same fitting approach as for the curves recorded under different salt concentrations (*k*_T_, *k*_isc_*k*_–_, and *Q* fixed and *k*_+_ fitted individually) yielded fitted curves well in agreement with the measured TRAST curves and *k*_+_ rates, which significantly increased with higher amounts of positively charged lipid head groups in the membranes (Figure 4B with inset B1).

Given the prominent dependence of the doublet state formation on pH, we next investigated if also the local buffer conditions at the membranes could be reflected in the TRAST curves, as exploited in proton-exchange studies by FCS using pH-sensitive fluorophores and monitoring the exchange rate of protons to and from these fluorophores.^22,23^ More specifically, we investigated if local buffering effects can be observed via the hydroxyl ion-dependent transitions to and from the NAO doublet radical state. First, for POPC vesicles with NAO in different concentrations of HEPES buffer (pH fixed at 7.4), clear effects on the recorded TRAST curves could be observed (Figure 4C). Since the singlet–triplet state transitions are not expected to depend on the buffer concentration, we fixed *k*_T_, *k*_isc_, and *Q* in the parameter fitting to values as determined above, but let *k*_+_ and *k*_–_ be fitted individually to each of the curves. This generated fitted curves well in agreement with the experimental TRAST curves and with both *k*_+_ and *k*_–_ monotonously increased with increasing HEPES buffer concentrations (insets C1 and C2, Figure 4C). Similar effects were also found with different phosphate buffer concentrations added (Supporting Information, Figure S8). Next, we investigated the influence of buffering lipid head groups (POPA) at the membrane surface. In TRAST curves recorded from POPC vesicles with increasing molar fractions of POPA, a prominent effect was found, with a noticeably faster relaxation of the doublet state radical population (Figure 4D). The same fitting approach as for the bulk buffer measurements (*k*_T_, *k*_isc_, and *Q* fixed, *k*_+_ and *k*_–_ fitted individually) could well reproduce the experimental curves, and as for bulk solution buffer, both *k*_+_ and *k*_–_ increased monotonously with higher molar fractions of POPA in the SUVs (insets D1 and D2, Figure 4D). Thus, like proton-exchange measurements with FCS,^22,23^ TRAST measurements on NAO, following the hydroxyl ion-dependent rates *k*_+_ and *k*_–_, allow monitoring of local buffering properties, either from a three-dimensional (3D) bulk buffer above the membrane or from a two-dimensional (2D) buffer at the membrane surface itself. It can be noticed that the increase in *k*_+_ with increased buffer concentrations (in bulk or at the membrane) is more prominent than for *k*_–_. One possible reason for this is that *k*_+_ is more directly related to the local concentration and interaction with the hydroxyl ions. For the back-reaction from the doublet radical state (*k*_–_) to occur, the radical must first react with a hydroxyl radical ion and form a complex, before a hydroxyl ion is released (Figure 3A).

### Vesicles with Cardiolipin (CL) and Live-Cell Measurements

**Figure 5:**
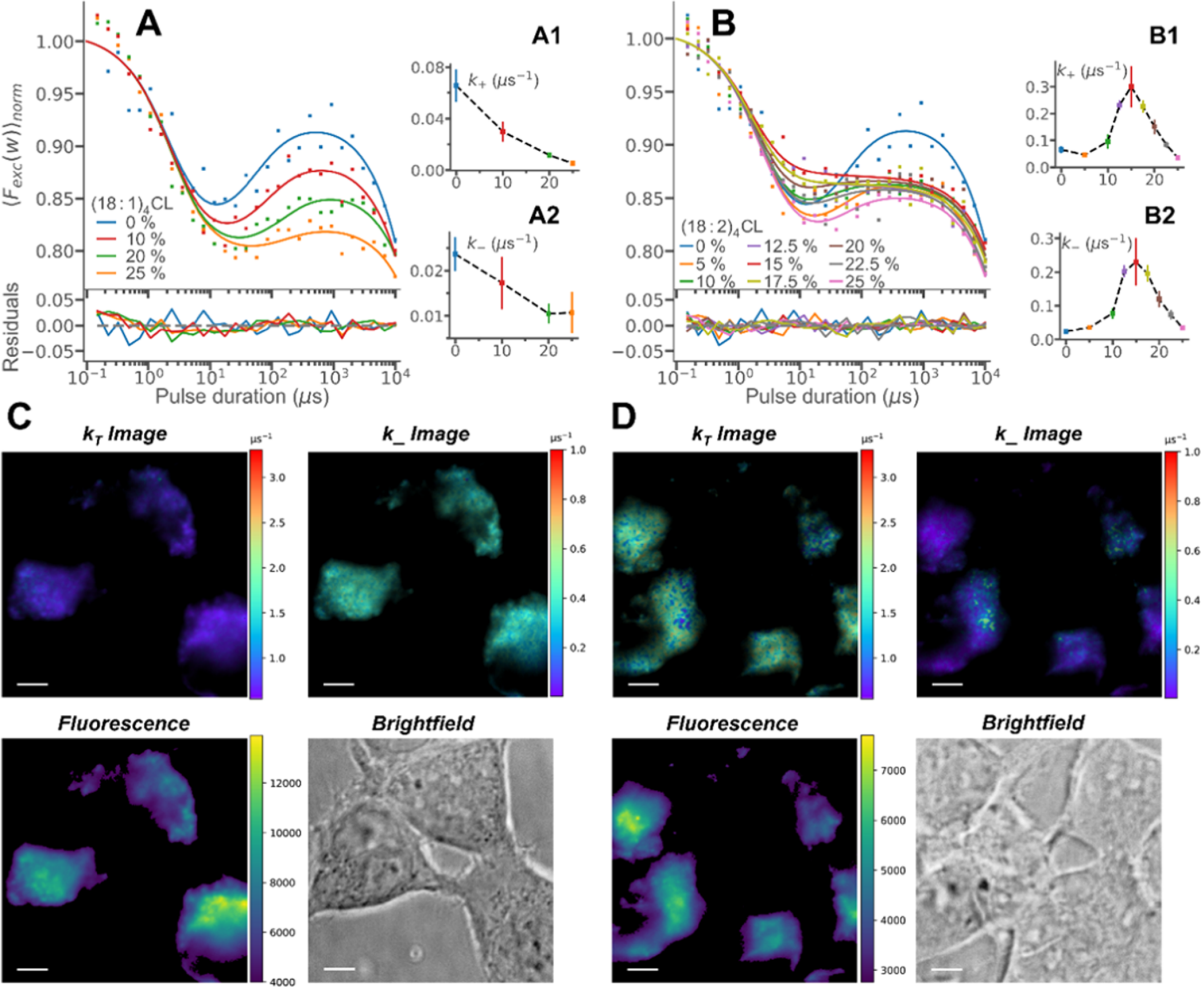
Experimental TRAST curves from vesicles with Cardiolipin (CL) and live-cell measurements (in DPBS buffer, pH 7.4, air atmosphere, *I*_exc_: 11.7 kW/cm^2^). TRAST curves were fitted globally using the photophysical model of Figure 3B, see the main text. (A) TRAST curves were recorded from vesicles with different molar fractions of (18:1)_4_CL. Individually fitted *k*_+_ and *k*_–_ rates are shown in insets A1 and A2. (B) TRAST curves recorded from vesicles with different molar fractions of (18:2)_4_CL, with the individually fitted *k*_+_ and *k*_–_ rates in insets B1 and B2. (C) Upper line: TRAST images of HEK293A cells, showing the *k*_T_ and *k*_–_ rates of added NAO. Lower line: Corresponding fluorescence intensity (left) and bright-field (right) images of the cells. (D) Corresponding images as in 6C, recorded from HEK293A cells incubated with 20 μM 16-doxyl. Scale bars: 5 μm.

Given the strong binding of NAO to CL, as a basis for its use as a mitochondrial marker, we recorded TRAST curves of POPC vesicles with different amounts of two different CL lipids: (18:1)_4_CL and (18:2)_4_CL, differing only in the number of double bonds in the acyl chains. Figure 5A shows TRAST curves recorded from vesicles with different concentrations of (18:1)_4_CL added. With increasing (18:1)_4_CL concentrations, the doublet radical state population is lowered and its relaxation slowed down. No effect on the singlet–triplet state transitions is evident. Based on these observations, we applied a fitting approach with *k*_T_, *k*_isc_, and *Q* fixed to their values as determined above (Supporting Information, Table S1) and with *k*_+_ and *k*_–_ fitted individually, which could well reproduce the experimental TRAST curves (Figure 5A, insets A1 and A2). The fitted *k*_+_ rates decreased significantly with higher (18:1)_4_CL concentrations. A slight decrease was also obtained for the fitted *k*_–_ rates. The prominent decrease in *k*_+_ can likely be attributed to the negative charge of CL, which counteracts the binding of OH^–^ to NAO and thus reduces the formation of ^3^[NAO^+•^OH^–^] complexes, which precedes the formation of NAO doublet radicals (Figure 3A). Interestingly, the effects seen on TRAST curves recorded from vesicles with (18:2)_4_CL (Figure 5B) were quite different from those with (18:1)_4_CL. Here, we observed a reduction in the doublet radical state build-up for added (18:2)_4_CL concentrations up to 15 mol % but then a slight increase in the build-up when even higher concentrations were added. The same fitting approach as used for the curves in Figure 5A (*k*_T_, *k*_isc_, and *Q* fixed to the values given in the Supporting Information, Table S1, and *k*_+_ and *k*_–_ (and *k*_B_) fitted individually) could reproduce also these experimental TRAST curves (Figure 5B). Upon adding (18:2)_4_CL, the fitted *k*_+_ and *k*_–_ rates first increased up to a distinct maximum at an (18:2)_4_CL concentration of 15 mol % and then decreased to the same levels as in the absence of (18:2)_4_CL (insets B1 and B2, Figure 5B). This observation likely reflects that a phase transition takes place at around 15 mol %, as has been found to occur differently for (18:1)_4_CL and (18:2)_4_CL.^2^

Finally, following TRAST measurements of NAO on vesicles in solution, we next set out to monitor the same transitions, in membranes of live cells. NAO was added to Hek293 cells, at concentrations of 250 nM, which were low enough not to show any indications of dimer formation (from the intensity ratio between the green and red emission channels). Dual-color imaging of NAO, together with the mitochondrial dye Mitotracker Red, showed strong co-localization of the dyes, indicating that NAO also at this concentration preferentially binds to CL and to the mitochondrial membranes of the cells (Figure S9).

First, TRAST curves were generated from the total fluorescence recorded from all cells in the images, acquired with different excitation pulse widths applied (Figure S10). These curves were very similar to the curves recorded from POPC vesicles with 15% (18:2)_4_CL (Figure 5B). The type of acyl chains present in CL varies between different species. Interestingly though, this resemblance is well in agreement with (18:2)_4_CL being the predominant CL in mammalian cells^10^ and having a typical concentration of 15% in the mitochondrial membranes.^1^ TRAST curves were then recorded from the cells, after incubation with different concentrations of 16-doxyl (0–75 μM) added (Supporting Information, Figure S10), and they showed the same effects on *k*_*T*_ and *k*_–_ upon addition of 16-doxyl as found in the vesicle measurements (Supporting Information, Figure S11A). TRAST images of the cells were then generated, as previously described,^30^ in this case using a conversion table, directly relating the excitation irradiance and the measured decay and rise amplitudes (the dark and doublet radical state populations) to determine *k*_T_ and *k*_–_ (other parameters fixed, see the Methods section). Thereby, rate images of the cells could be readily computed (Figure 5C,D).

In Figure 5C, the *k*_–_ rates imaged in the cells are similar to the values found in the measurements of POPC vesicles with 15% (18:2)_4_CL (Figure 5B). With 16-doxyl added into cells (Figure 5D), we also here found a significant decrease in the *k*_–_ rates, and an increase in the *k*_T_ rates, quite in agreement with the whole-cell (Figure S10A) and vesicle (Figure S11) measurements. In the cellular images of the *k*_T_ and *k*_–_ rates (Figure 5C,D), some spatial variations in the rates can be noted, showing that the rates can be imaged in a spatially resolved manner and possibly reflecting different oxygenation and redox conditions close to the mitochondria.

## Discussion

By TRAST measurements, supported by computational simulations, we show that the mitochondrial localization fluorophore NAO exhibits prominent singlet–triplet state transitions and can act as a light-induced Lewis acid forming a red-emissive doublet radical. In SUVs, we systematically studied how different microenvironmental conditions affected the blinking properties of NAO due to these transitions. With a simple photophysical model, and using global parameter fitting with a limited number of free parameters, we could reproduce the experimental TRAST curves in a robust manner. These studies show that the blinking properties of NAO can be monitored under biologically relevant conditions and that they are highly environment-sensitive, specifically reflecting local oxygen concentrations, redox conditions, membrane charge and lipid head group compositions, pH, and buffer capacity. These parameters are of large importance for oxidative phosphorylation, suggesting that NAO, by its fluorescence blinking properties, can be turned into a multifunctional sensing probe for inner mitochondrial membrane and bacterial membrane studies. We also show that the blinking properties can be imaged in live cells, in a spatially resolved manner, with the NAO dye reflecting the conditions on site, at the mitochondrial membranes. Since the blinking properties of NAO are sensitive to a large number of parameters, it may be difficult to analyze them separately in live cells and tissues, but relative changes can be followed providing useful metabolic and microenvironmental information. At the same time, the blinking properties of NAO offer independent and essentially orthogonal information to conventional fluorescence readouts, and the blinking properties can also be quite differently affected by the environment from one type of fluorophore to another. In this respect, the use of multiple fluorophore probes at different wavelengths, with different dependencies on membrane conditions, is likely an interesting strategy to add detail and specificity. At the NAO concentrations used in this study, no characteristic red (640 nm), excimer fluorescence of π–π-stacked NAO dimers was found, indicating that the concentrations of NAO were too low for NAO to bind both the two phosphate residues of CL in CL-containing membranes. This may reduce the specific affinity enhancement of NAO to CL, attributed to electrostatic interaction between adjacent NAO molecules bound to the same CL.^16^ Thus, some binding to anionic phospholipids in general can also be expected. However, co-localization studies with another mitochondrial localization dye still clearly indicated that NAO preferentially binds to CL in the IMMs. Moreover, any perturbations in the membranes caused by the high concentrations required for excimer formation will be minimized, and the TRAST measurements are also not limited to a relatively narrow concentration range of NAO in the membranes, as required for NAO readouts based on excimer fluorescence.^15^ Interestingly, quite different TRAST curves were recorded from SUVs with (18:1)_4_CL compared to SUVs with (18:2)_4_CL. This illustrates the important role of the CL acyl chain composition for the membrane properties, that the photophysical transitions observed in NAO are sensitive to these effects, and the ability of TRAST studies of NAO to reveal this phenomenon. TRAST measurements on NAO, following the hydroxyl ion-dependent rates *k*_+_ and *k*_–_, allow monitoring of local buffering properties, either from a 3D bulk buffer above the membrane or from a 2D buffer at the membrane surface itself. These measurements do not, in contrast to proton-exchange studies by FCS,^21−23^ require single-molecule detection conditions and thus open for more broadly applicable local proton-exchange and buffering studies. Taken together, singlet–triplet and photoinduced electron-transfer transitions in NAO, monitored by TRAST, open new possibilities for fundamental membrane studies, in artificial vesicles as well as in live cells. Local environmental information on molecular dynamics and interactions can thereby be obtained and imaged, which is difficult, if possible at all, to obtain by other means. This is enabled, not by the development of new spectacular fluorophore reporters, but by taking advantage of the fluorescence blinking properties of an established location-specific fluorophore, rendering it multiparametric sensing properties.

## Supporting Information Available

Electronic state model for NAO (Section S1); spatial distribution of excitation rates, calculation of average rates (Section S2); preparation of lipid vesicles (Section S3); cell preparation (Section S4); setup for TRAST measurements (Section S5); TRAST data analysis (Section S6); TRAST images of cells (Section S7); fluorescence lifetime measurements (Section S8); spectroscopic measurements (Section S9); computational details for the fluorophore simulations (Section S10); computational simulations (Section S11); fitting of TRAST curves Figure 2A–D (Section S12); effects of spin labels and redox environment (Section S13), and Figures S1–S11 and Table S1, as referred to in the main text (PDF)
