# Supplementary material for: Imaging Fluorescence Blinking of a Mitochondrial Localization Probe: Cellular Localization Probes Turned into Multifunctional Sensors
Source: J Phys Chem B. 2022 Apr 13;126(16):3048–58. doi: 10.1021/acs.jpcb.2c01271 (PMC9059120; doi:10.1021/acs.jpcb.2c01271)
Supplement: Supplementary file 1 — jp2c01271_si_001.pdf [file jp2c01271_si_001.pdf]

Imaging Fluorescence Blinking of a Mitochondrial Localization Probe - Cellular Localization Probes Turned into Multi-functional Sensors

Zhixue Du[a], Joachim Piguet[a,+], Glib Baryshnikov[b,+], Johan Tornmalm[a], Baris Demirbay[a], Hans Ågren[c], Jerker Widengren[a,*]

[a] Royal Institute of Technology (KTH), Experimental Biomolecular Physics, Dept. Applied Physics, Albanova Univ Center 106 91 Stockholm, Sweden

[b] Laboratory of Organic Electronics, Dept. Science and Technology, Linköping University, 601 74 Norrköping, Sweden

[c] Dept. Physics and Astronomy, Uppsala University, Box 516, 751 20 Uppsala, Sweden

[+] Contributed equally

* Corresponding author: Email: jwideng@kth.se, Phone: +46-8-7907813

Supporting Information:

S1. Electronic state model for NAO

Based on the electronic state model shown in Figure 3B, the electronic state population dynamics of a NAO^+ fluorophore, subject to a constant excitation photon flux of Φ_{exc} starting at time t=0, is given by

$$\frac{d}{dt}\bar{P}(t) = M \cdot \bar{P}(t) \tag{S1}$$

Here, $\bar{P}(t) = \left[[\,^1NAO^+](t), [\,^3NAO^+](t),\ [\,^2NAO^\bullet](t), [\,^BNAO](t) \right]^T$ represents the population probabilities of the ground and excited singlet states ($[\,^1NAO^+]$), the triplet state ($[\,^3NAO^+]$), the ground and excited doublet radical states ($[\,^2NAO^\bullet]$) and an irreversible dark state ($[\,^BNAO]$) at time t and

$$M = \begin{bmatrix} -k_{isc}' & k_T & 0 & 0 \\ k_{isc}' & -(k_T + k_+) & k_- & 0 \\ 0 & k_+ & -(k_- + k_B) & 0 \\ 0 & 0 & k_B & 0 \end{bmatrix} \tag{S2}$$

is the model matrix describing the transitions between the states. In the matrix, $k_{isc}'(\bar{r}) = k_{isc} \cdot (\sigma_1 \cdot \Phi_{exc})/(\sigma_1 \cdot \Phi_{exc} + k_{10}^1)$ is the effective intersysytem crossing rate from the singlet states to the triplet state, with σ_1 denoting the excitation cross section of the singlet ground state, $_0^1NAO$. The initial condition for Eq. (S1) is

$$\bar{P}(0) = [1\ 0\ 0\ 0]^T \tag{S3}$$

, assuming all NAO fluorophores are in the singlet (ground) state before onset of excitation at $t = 0$.

For a rectangular excitation pulse, Φ_{exc} is constant throughout the excitation duration and the matrix M is not time dependent. The general solution to Eq S1 is then

$$\bar{P}(t) = e^{Mt} \cdot \bar{P}(0) \tag{S4}$$

The dependence of the detected fluorescence at time, t, after onset of excitation is then given by

$$F(t) = {}^{1}q_F \cdot {}^{1}q_D \cdot k_{10} \cdot \frac{\sigma_1 \cdot \Phi_{exc}}{\sigma_1 \cdot \Phi_{exc} + k_{10}} \cdot [{}^{1}NAO^{+}](t) + {}^{2}q_F \cdot {}^{2}q_D \cdot k_{10} \cdot \frac{\sigma_2 \cdot \Phi_{exc}}{\sigma_2 \cdot \Phi_{exc} + k_{10}} \cdot [{}^{2}NAO^{\bullet}](t) \tag{S5}$$

, with σ_X denoting the excitation cross section, ${}^{X}q_F$ the fluorescence quantum yield and ${}^{X}q_D$ the detection quantum yield of the emission from the singlet (X=1) and doublet (X=2) state, respectively. For the excitation conditions in our study, $k_{10} \gg \sigma_1 \cdot \Phi_{exc}, \sigma_2 \cdot \Phi_{exc}$, so that we can assume

$$F(t) = {}^{1}q_F \cdot {}^{1}q_F \cdot \sigma_1 \cdot \Phi_{exc} \cdot \left([{}^{1}NAO^{+}](t) + Q \cdot [{}^{2}NAO^{\bullet}](t)\right) \tag{S6}$$

, with $Q = ({}^{2}q_F \cdot {}^{2}q_D \cdot \sigma_2)/({}^{1}q_F \cdot {}^{1}q_D \cdot \sigma_1)$ representing the relative brightness of the doublet state, ${}^{2}NAO^{\bullet}$, compared to the singlet state, ${}^{1}NAO^{+}$.

S2. Spatial distribution of excitation rates, calculation of average rates

The Gaussian shape of the excitation beam means that the excitation photon flux, $\Phi_{exc}(\bar{r})$, is a function of position in the sample. As a consequence, a detailed TRAST analysis should include a spatial dependence to both the excitation rates and the resulting electronic state populations. The total fluorescence signal on each pixel of the camera then becomes a convolution of $[{}^{1}NAO^{+}](t) + Q \cdot [{}^{2}NAO^{\bullet}](t)$ and the microscope collection efficiency function, $CEF(\bar{r})$, as shown in Eq. (4). However, simulating the whole 3D sample volume, and computing the projected 2D image on the camera, becomes a costly operation when performed in each iteration of the fitting algorithm. While this procedure is possible, and sometimes required, we found that pre-computing an average observed excitation rate, $\hat{k}_{01}$, for each pixel or ROI to be analyzed, speeds up the fitting significantly, without appreciable loss of accuracy. The approximate $\hat{k}_{01}$ is computed once, before fitting starts, by weighting $k_{01}(\bar{r})$ by brightness and collection efficiency, $CEF(\bar{r})$, in the following manner

$$\hat{k}_{01} = \frac{\iiint k_{01}(\bar{r}) \cdot \hat{S}_1(\bar{r}) \cdot CEF(\bar{r})\, dV}{\iiint \hat{S}_1(\bar{r}) \cdot CEF(\bar{r})\, dV} \tag{S7}$$

$\hat{S}_1(\bar{r}) = k_{01}(\bar{r})/(k_{10} + k_{01}(\bar{r}))$ represents the population of ${}^{1}_{1}NAO^{+*}$ at onset of excitation, after equilibration between the singlet states ${}^{1}_{0}NAO^{+}$ and ${}^{1}_{1}NAO^{+*}$, but before build-up of the other states.

S3. Preparation of lipid vesicles.

All lipids (POPC, POPG, POPA, DOTAP, 18:1 Cardiolipin, 18:2 Cardiolipin (Heart, Bovine)) were purchased from Avanti Polar Lipids, Inc. 1.74 umol lipid chloroform solution was dried under a gentle flow of N_2. After complete removal of any residual solvent, 1.4 ml DPBS buffer (pH 7.4, Sigma-Aldrich, cat. no. D8537) was added to the dried lipid and vortexed for 1 min. The resulting solution was sonicated at 0 ^{O}C for 5min by a Branson SFX250 sonicator at 50% duty cycle (0.50 s on/off), 50% power (125 W) and a 1/8” microtip (Emerson Electric Co, St. Louis, MO, USA). After sonication, the solution was centrifuged for 15min at 14000g,

and the supernatant was filtered using a 0.2 um spin-filter (Corning, NY, USA) to remove large aggregates. The Doxyl containing vesicles were made by adding 16-DOXYL stearic acid (Sigma-Aldrich) directly to the final vesicle solution and incubated for 5 min. Before fluorescence detection, 250 nM NAO (Sigma-Aldrich) was added to the final vesicle solution and incubated for 10 min.

S4. Preparation of cells.

HEK293A cells were maintained in DMEM medium supplemented with 10% fetal bovine serum (Life Technology), 1% penicillin-streptomycin. Before TRAST measurements, cells were grown in glass-bottom 8-well cell culture plates (Nunc Lab-Tek II Chambered Coverglass) for 1-2 days. Thereafter the cell culture medium was replaced with DPBS buffer (Sigma-Aldrich), and 16-DOXYL stearic acid (Sigma-Aldrich) was added to the buffer. Cells were incubated 15 minutes at 37°C, and then NAO was added and incubated for another 15 minutes.

S5. Experimental setup for TRAST measurements.

TRAST measurements were carried out on a home-built TRAST setup (Figure 1A) based on an inverted epi-fluorescence microscope (Olympus, IX73). Fluorescence is excited by a 488 nm diode laser (Cobolt, 06-MLD, 200 mW) using an excitation filter (Semrock BrightLine 488/10). The laser beam was modulated by an acousto-optic modulator (AOM; AA Opto Electronics, MQ180-A0,25-VIS). The expanded laser beam was defocused by a convex lens, reflected by a dichroic mirror ((FF506-Di03, Semrock)) and then focused close to the back aperture of the objective (Olympus, UPLSAPO 60x/1.20 W) to produce a wide-field illumination in the sample (beam waist ω_0=15µm (1/e^2 radius)). The fluorescence signal was collected by the same objective, passed through the same dichroic mirror, split into two detection channels by a dichroic mirror (T647lpxr, Chroma), then passed though double emission filters ((BrightLine 530/55, Semrock) for the green channel and one emission filter (ET670/50m, Chroma) for the red channel to remove scattered laser light, and was then fed to two separate sCMOS cameras (Hamamatsu ORCA-Flash4.0 v2). The experiments were controlled and synchronized by custom software implemented in Matlab. A digital I/O card (PCI-6602, National Instruments) was used to trigger the camera and generate random excitation pulse trains sent to the AOM driver unit. For experiments with modified oxygen concentrations, a stage incubator system (WP and FC-7, Chamlide, Live Cell Instruments) was used.

S6. TRAST data analysis.

The TRAST data was analyzed using a software implemented in Matlab, as previously described [1]. The recorded TRAST data was first pre-processed by subtraction of the static ambient background, optional binning to either larger pixels or regions of interest (ROIs) within the recorded images, and correction for bleaching. The bleaching correction was based on 10 reference frames, recorded in between the regular frames throughout the measurements (see TRAST spectroscopy above). Moreover, by discarding the data

from the first 3-4 excitation pulse trains, a close to steady-state could be established. The overall bleaching was then maximally 5-10 % of the total detected intensity in the vesicle experiments. In the cell measurements, where fluorophores were not replenished by vesicle diffusion, the bleaching was higher, in rare cases as high as 50 %, but a similar steady-state as in the vesicle experiments was nonetheless obtained after 3-4 excitation pulse trains.

In all measurements, TRAST curves were produced by calculating $\langle F_{exc}(w)\rangle_{norm}$ within a region of interest (ROI) corresponding to a $15\ \mu\text{m}$ radius in the sample plane for both the vesicle and live cell measurements, centered on the excitation beam. In both cases, fitting of photophysical rate parameters was then performed by simulating theoretical TRAST curves using Eqs . (1-3) and comparing them to the experimental data. The simulations also used an adjusted value for the pulse widths, *w*. A small but constant shift in pulse duration, Δw, caused by non-zero rise- and fall-times of the AOM, lead to a relative error in illumination time given by $\Delta w/w$. With Δw in the order of 10 ns, and constant for all pulse widths, this effect is only relevant for the shortest pulses. The set of rate parameters best describing the experimental data was then found using non-linear least squares optimization, based on the Levenberg-Marquardt algorithm, and using Matlab´s Optimization Toolbox with its standard convergence criteria. In the fit, the excited state lifetimes of ${}^1_1NAO^{+*}$ and ${}^2_1NAO^{\bullet *}$ were fixed to 1.6 ns and 4.6 ns, as found from time-correlated single photon counting measurements (see Results), and such that $1/\tau_f = k_{10} + k_{isc}$ in the case of ${}^1_1NAO^{+*}$. Relative differences in the excitation rates between the doublet and singlet states are accounted for in the relative brightness parameter, *Q* (Eq. 1), and an average singlet excitation rate, $\hat{k}_{01}$, was calculated for each pixel or ROI using Eq. (S7) (see SI for details) and an excitation cross section for ${}^1_0NAO^{+}$ of $\sigma = 20.8 \cdot 10^{-17}\ \text{cm}^2$ [2].

For each of the environmental conditions/vesicle samples studied at least three separate TRAST curves were recorded. For each condition of cell sample studied, typically six to eight separate TRAST curves were recorded. Error limits of the determined parameter values were calculated as the standard error of the mean, with one parameter at a time kept as a free parameter in the fittings of the TRAST curves.

S7. TRAST images of cells.

Images of k_T and k_- rates were determined from pixel-wise averaged fluorescence intensity values, acquired upon excitation with pulses within three different pulse width ranges; $F(w_{short})$: -100 ns, $F(w_{\mu s})$: 1 µs - 10 µs (2.4, 3.5625 and 5.3 µs), and $F(w_{ms})$: 0.1 ms - 1 ms (126.9, 188.737 and 280.725 µs). From these values, two difference values ($D_1 = F(w_{short}) - F(w_{\mu s})$ and $D_2 = F(w_{ms}) - F(w_{\mu s})$) were then obtained. Before calculating D_1 and D_2, the pixel-wise fluorescence intensity values of the images were corrected for static ambient background and photobleaching, following the procedure described above. The resulting fluorescence intensity of each image was then normalized and corrected with an instrument response function, similarly as for the vesicle measurement data. To improve photon statistics and minimize effects of stray photons, images were filtered with a 3x3 pixel median filter. In addition, the median value for each pixel, recorded over the sequence of images within each excitation pulse width range, was computed to give

a median normalized intensity value for the corresponding pulse width range. The fluorescence intensity differences were then calculated from these normalized values.

The local excitation rate, k_{01}, was determined for each pixel, as previously described.[30] With knowledge of the total laser power onto the sample, the laser excitation intensity distribution was determined from the image of a fluorophore (Rhodamine 6G) solution, recorded at non-saturating excitation conditions. Each pixel of the reference image was then converted into a local irradiance value and the excitation rates at each location were calculated from the excitation cross-sections σ_1 and σ_2. To convert the intensity difference value measured for each pixel to rates, a conversion table was pre-calculated based on the photophysical model of NAO (Figure 3B), and with rate parameters determined following the same procedure as in the vesicle measurements (Figure S11A), with addition of 16-Doxyl to the membranes only affecting the rates k_T and k_-, and with all other rates kept constant. The conversion table consists of all D_1 and D_2 values obtained from simulations, based on the model in Figure 3B and the corresponding equations (Supplementary, part S1), covering sets of parameter values, of k_{01} ranging between 0.1 and 20 μs^{-1}, k_- ranging between 0.001 and 2 μs^{-1} and k_T ranging between 0.1 and 6 μs^{-1}. The final rate images were then determined from the experimentally determined difference images, the local excitation rate image and this conversion table.

S8. Fluorescence Lifetime measurements.

Time-correlated single photon counting (TCSPC) lifetime measurements were performed on POPC lipid vesicle solutions, prepared as described above, and then labeled with 500 nM NAO. A pulsed diode laser (485 nm, Picoquant GmbH, LDH-D-C-485) was used for excitation, fed into an epi-illuminated microscope (Olympus FV1200) with a water immersion objective (60x, NA1.2, Olympus, UPlanSApo). The fluorescence decay was detected by single-photon counting avalanche photodiodes (Picoquant, Tau-spad in the red channel and Perkin & Elmer, SPCM-AQR-14 in the green channel), using appropriate emission filters (Chroma, HQ535/70 and HQ720/150) in front of the detectors. The signals were fed into a data acquisition card (Hydraharp, Picoquant GmbH), and then fit to a double exponential decay based on non-linear least squares minimization (Symphotime, Picoquant GmbH). Due to the much higher signal/noise ratio in the green detection channel, data from this channel was used for the fluorescence lifetime analyses. However, changes in the amplitudes of the short- and long lifetime components can be expected to follow the same trends as those in the red channel.

S9. Spectroscopic Measurements.

Absorption spectra of SUVs labeled with NAO, prepared as described above, were measured with a spectrophotometer (UV5, Mettler Toledo), emission spectra with a spectrofluorometer (FluoroMax-3, HORIBA), using an excitation wavelength of 488nm with a 1nm bandpass slit.

S10. Computational details for the fluorophore simulations.

Initially, the singlet ground state of the AO, NAO and $[NAO{\cdot}OH]^-$ species was optimized by the M062X/6-31+G(d,p) method [3-6]. The solvent effect was considered at the Polarizable Continuum Model (PCM) level [7] using the Solvent Model Density (SMD) variant of the integral equation formalism (IEFPCM) [8] and demonstrated a good performance in computations of acidity constants for various types of organic acids and bases [9, 10]. The first excited triplet state of AO, NAO and $[NAO{\cdot}OH]^-$ as well as the doublet ground state, ${}^2_0NAO^{\bullet}$ were optimized within the unrestricted Kohn-Shem formalism at the same M062X/6-31+G(d,p) level of theory accounting also for the SMD model. At the same time, the lowest excited singlet state for all studied structures was optimized by the spin-restricted time-dependent (TD) DFT method [11] by using the same M062X/6-31+G(d,p) level with the SMD approach. Molecular electrostatic potential (MESP) plots for the singlet and triplet states of NAO (${}^1_0NAO^+$, ${}^1_1NAO^{+*}$, ${}^3NAO^+$) were then generated at the same level of theory in order to find the most attractive centers for OH^- attachment. The pK_a values for the AO and NAO^+ species were computed by a standard procedure [9] by means of the following protolithic equilibria: AO + H^+ $\leftrightarrow$ AOH^+ and NAO^+ + H^+ $\leftrightarrow$ $NAOH^{2+}$ accounting for the two protonation centers in the AO dye (intracyclyc (>N) and exocyclic ($-N(CH_3)_2$) nitrogen atoms) as well as in the NAO^+ dye (intracyclyc (>N-C_9H_{19}) and exocyclic ($-N(CH_3)_2$) nitrogen atoms). All the calculations were performed with the Gaussian 16 software [https://gaussian.com/citation/].

S11. Computational simulations of the photophysical transitions of NAO

To add evidence and further investigate the prerequisites for NAO to act as a light-induced Lewis acid, computational modelling was performed (see Section S10 for methodological details). First, pK_a values were calculated, for NAO as well as for AO, serving as a reference molecule having the same chromophore structure as NAO. For AO, our calculations could reproduce flash photolysis and fluorometry data on this dye [12], with a higher pK_a calculated for the S_1 state (pK_a=16.4) than for the S_0 state (pK_a=12.3). For the T_1 state however, our calculations predict even stronger basic properties (pK_a=17.8) than for the S_1 state. The calculated pK_a values for S_0, S_1 and T_1 states of the AO dye correlate well with the negative Mulliken charges on the intracyclic (>N) nitrogen atom (-0.35*e*, -0.38*e* and -0.51*e*, respectively). This confirms that the AO dye acts as a photo-base after excitation into the S_1 state, but also predicts, in some disagreement with previous experimental findings [12], that the T_1 state of AO has stronger basic properties than the S_0 state.

For AO acting as a photo-base, its intra-cyclic nitrogen atom has been identified as the site of the protonation [12]. NAO on the other hand, is typically found in its NAO^+ cationic form, and its intracyclic nitrogen atom is bound to an *n*-nonyl group, which is then not available as a site of protonation. In agreement with this, our calculated pK_a values for NAO were found to be highly negative: -21.0 (S_0), -9.5 (T_1) and -10.5 (S_1). Similarly, the exocyclic nitrogen atoms ($-N(CH_3)_3$ groups) for both the AO and the NAO^+ dyes also do not demonstrate properties of a base, but rather strong acidity, with calculated pK_a values which are negative or close to zero: S_0(AO)/(NAO) -4.4/-3.3, S_1(AO)/(NAO^+) -4.2/-3.0, T_1(AO)/(NAO^+) 0.11/0.14.

To be able to account for the pH dependence for the formation of the red emissive species as seen in the

TRAST curves (Figure 2D), we next investigated the prerequisites for NAO to act as a Lewis acid, capable to accept an electron pair from an OH^- anion. First, we considered different centres of the NAO^+ cation for attachment of OH^- anions, based on molecular electrostatic potential (MESP) calculations (Figure S5A). We then found that in-plane OH^- interactions with the aromatic, methyl and nonyl protons of NAO^+ lead to stable H-bonded complexes $[\mathrm{NAO}^+ \cdot \mathrm{OH}^-]$ in the ground S_0 state as well as in the excited T_1 and S_1 states. However, no evident charge redistribution occurred in such $[\mathrm{NAO}^+ \cdot \mathrm{OH}^-]$ complexes, compared with the non-coupled OH^- and NAO^+ counterparts. On the other hand, positioning OH^- over the NAO plane (over the inner pyridine ring where the area of positive MESP is clearly pronounced in the T_1 state, but not in the S_1 or S_0 state, Figure S5A) lead to the formation of a weakly bound $[\mathrm{NAO}^+ \cdot \mathrm{OH}^-]$ complex in the ground singlet state (E_{bind} = 2 kcal/mol^{-1}). Optimization of this complex in the T_1 excited state ($^3[\mathrm{NAO}^+ \cdot \mathrm{OH}^-]$) lead to a slightly endothermic formation of a charge-transfer complex $^3[\mathrm{NAO}^\bullet\ \mathrm{OH}^\bullet]$. This actually corresponds to two coupled doublet state radicals (Figure S5B), formed in a photo-induced intermolecular electron transfer, or simply a photo-reduction, process [13].

The slightly endothermic dissociation of the $^3[\mathrm{NAO}^\bullet\ \mathrm{OH}^\bullet]$ complex can then lead to a new type of NAO radical species, a neutral radical in a ground doublet state, $^2NAO^\bullet$. Our calculations indicate that $^2NAO^\bullet$ can absorb and emit light in the low-energy region ($E({}^2_0NAO^\bullet - {}^2_1NAO^\bullet)_{vert}$: 559 nm, $f({}^2_0NAO^\bullet - {}^2_1NAO^\bullet)$: 1.2×10^{-3}, $E({}^2_1NAO^\bullet - {}^2_0NAO^\bullet)_{vert}$: 861 nm, $f({}^2_1NAO^\bullet - {}^2_0NAO^\bullet)$: 7.7×10^{-3}, where f denotes the oscillation strength of the electronic transition. The computational calculations thus support the view that upon onset of excitation in the TRAST measurements, and following $^1NAO^+ \rightarrow {}^3NAO^+$ inter-system crossing, side-track formation of a $^3[\mathrm{NAO}^\bullet\ \mathrm{OH}^\bullet]$ complex can take place. Dissociation of this complex can then subsequently lead to the generation of doublet radicals, $^2NAO^\bullet$. This $^2NAO^\bullet$ species is responsible for the long-lifetime fluorescence component, as observed experimentally (Figure S2), and as predicted from rate parameter calculations [14]. The computational calculations are also consistent with the observed increase in this component with higher I_{exc} (Figure 2C).

S12. Fitting of experimental TRAST curves measured in the different emission bands, and under different oxygen concentrations, excitation intensities and pH, to evaluate the photophysical model of Figure 3.

To evaluate the photophysical model, we first fitted the experimental TRAST curves measured in the different emission bands, and under different oxygen concentrations, excitation intensities and pH (Figures 2A-D). Several of the fitted parameter values obtained were then used and kept fixed in the fitting of the subsequent experimental TRAST curves. They are summarized in Table S1. First, the TRAST curves recorded at different pH (Figure 2D) were fitted. Since Q, k_{isc}, k_T, and k_- are not expected to vary with pH, these parameters were fitted globally (to the same values for all TRAST curves in the figure), while k_+ and k_B were fitted individually for each of the curves. This fitting could well reproduce the experimental curves, with Q and (k_{isc}, k_T, k_-) fitted to 0.4 ± 0.011 and (16.2 ± 2.8, 0.58 ± 0.16, 0.042 ± 0.0079) μs^{-1} and with k_+

displaying a prominent increase with increasing pH, from 0.021±0.0034 μs^{-1} up to 0.50±0.11 μs^{-1} (Figure S6), in agreement with the model and with k_+ being promoted by hydroxyl ions. k_B was fitted to $(7.2 \pm 1.1) \times 10^{-5}$ μs^{-1} at pH7.4, accounting for photobleaching at a time scale much slower than the other rates in the model. Next, the TRAST curves measured in different emission bands (Figure 2A) were fitted, with the rate parameters k_{isc}, k_T, k_+ and k_- fixed to the fitted values in Figure 2D, k_B fitted globally, and the relative brightness parameter *Q* fitted individually to each curve. The fitted curves well reproduced the experimental TRAST curves, with *Q* fitted to 0.26 ± 0.028, 0.32 ± 0.0087 and 0.36 ± 0.0050, the higher the fitted values the more red-shifted the emission filter used, and consistent with a doublet radical with red-shifted emission (Figure 2A). In the fitting of the TRAST curves recorded at different excitation intensities (Figure 2C), *Q*, k_+ and k_- were fixed to the fitted values in Figure 2D, k_{isc} and k_T were fitted globally for all curves, and k_B fitted individually to each curve. The fitted parameters were found to be close to the ones fitted to the TRAST curves in Figure 2D, with k_{isc} and k_T fitted to 15.6 ± 3.0 and 0.40 ± 0.053 μs^{-1}, respectively. Finally, TRAST curves measured under different oxygenation conditions (Figure 2B) were fitted, with *Q* and k_+ fixed to the same values as in Figure 2D, while a dependence on the oxygen concentration, [O_2], can be expected for the other fitting parameters (k_{isc} , k_T, k_- and k_B), which were therefore fitted individually to each of the curves. The fitted k_{isc} and k_T values displayed a clear increase with increasing [O_2] (Figure S7), in agreement with previous studies of other fluorophores [15]. For k_-, lower rates were found in the presence of oxygen (Figure S7), which is consistent with oxygen annihilating hydroxyl radicals, the latter promoting the recovery of NAO doublet radicals back into a singlet (or triplet) state form. k_B was fitted to ~1.4×10^{-4} μs^{-1}, irrespective of [O_2], and for all TRAST curves in this study, the obtained k_B values were found not to vary significantly. In the fittings, they were at least two orders of magnitude lower than the other fitted rate parameters and did not significantly influence these parameters, and we therefore do not further discuss the fitted k_B values.

S13. Effects of spin labels and redox environment.

In FCS and TRAST measurements addition of paramagnetic spin labels can be clearly observed to enhance the transitions between fluorophore singlet and triplet states, in solution as well as in biological membranes [1, 16]. We added the spin label doxyl in different concentrations (0 to 100 μM) into solutions containing POPC SUVs labelled with NAO, to see if similar effects could be observed also for this dye. Recorded TRAST curves (Figure S11A) showed decreased triplet state amplitudes with increasing concentrations of doxyl added, while the doublet state amplitudes remained essentially the same. With k_{isc} , k_+, and *Q* fixed to values as determined above, k_T and k_- were individually fitted and could well reproduce the recorded TRAST curves (Figure S11A, insets). The k_T rate was found to increase with higher doxyl concentrations, consistent with doxyl acting as a triplet state quencher, thereby also decreasing the overall formation rate of doublet radicals. In contrast, k_- was found to decrease upon adding doxyl, which can be attributed to the lower concentrations of hydroxyl radicals then being formed, and to doxyl in addition acting as a radical scavenger.

Since the relative decreases of the overall formation rate and of the k_- rate were similar, the population of the doublet radical state did not change significantly upon addition of doxyl. The effects of doxyl on the k_T and k_- of NAO are thus as prominent as those previously found for the deactivation rates of triplet and photo-oxidized states of rhodamine dyes, which can be used for low-frequency collisional interaction or compartmentalization studies in biological membranes and cells [1, 16].

To investigate effects on the transitions from the redox environment, we first added sodium ascorbate (NaAc) in different concentrations (0-2mM) to the same POPC vesicles with NAO. FCS and TRAST measurements have shown that addition of ascorbate can strongly influence dark state transitions of many fluorophores, promoting recovery of photo-oxidized fluorophores and thus enhancing fluorescence emission, but also enhancing photo-reduction [17]. For NAO, we found that when NaAc was added the population of doublet radicals increased, while no effect was noticed on the triplet state kinetics (Figure S11B). With k_T, k_{isc} , k_+, and Q fixed to values as determined above, and with k_- and k_B fitted individually to each of the curves, the measured TRAST curves could be well reproduced (Figure S11B). Similar to doxyl, the k_- rate was found to decrease with higher NaAc concentrations (Figure S11B, insets), consistent with ascorbate donating electrons and acting as a scavenger of hydroxyl radicals, which are needed for the deactivation of the doublet state radicals. Second, we investigated the effects of adding hydrogen peroxide (H_2O_2), which is formed in the mitochondria upon oxidative phosphorylation. In the TRAST measurements, addition of H_2O_2 was found to significantly decrease the doublet state population, as opposed to NaAc, but also had no effect on the triplet state population kinetics (Figure S11C). Having k_+, k_T and Q fixed to values as determined above, with k_{isc} fitted as a global parameter to the TRAST curves, and k_- fitted individually to each of the curves, generated fitted curves well in agreement with the measured TRAST curves (Figure S11C). While the fitted k_{isc} (20.2 ± 2.7 μs^{-1}) was well in line with previously determined rates, the k_- rate was found to increase linearly with higher H_2O_2 concentrations, in contrast to addition of NaAc, but concomitant with the decrease in the doublet state population. As an oxidant and a source of hydroxyl radicals H_2O_2 here promotes the k_- rate (Figure S11C, inset).

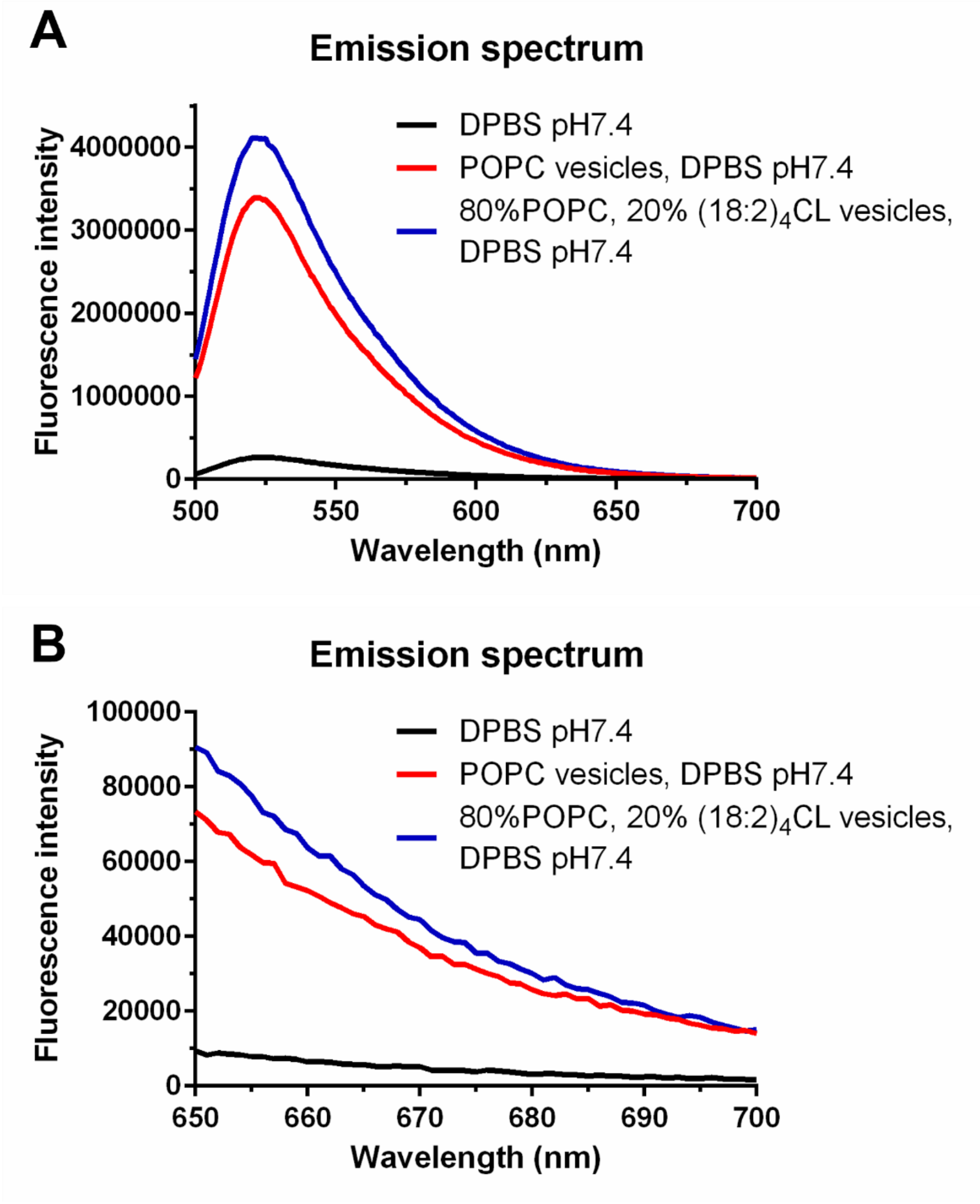

Figure S1. *Comparison of the emission spectrum recorded from 250nM NAO in DPBS buffer (Sigma-Aldrich, cat. no. D8537, pH 7.4, air atmosphere) with SUVs (different composition) present or not. (A) Emission spectrum from 500nm to 700nm. (B) Zoom-in of the emission spectrum in (A), in the range 650nm to 700nm.*

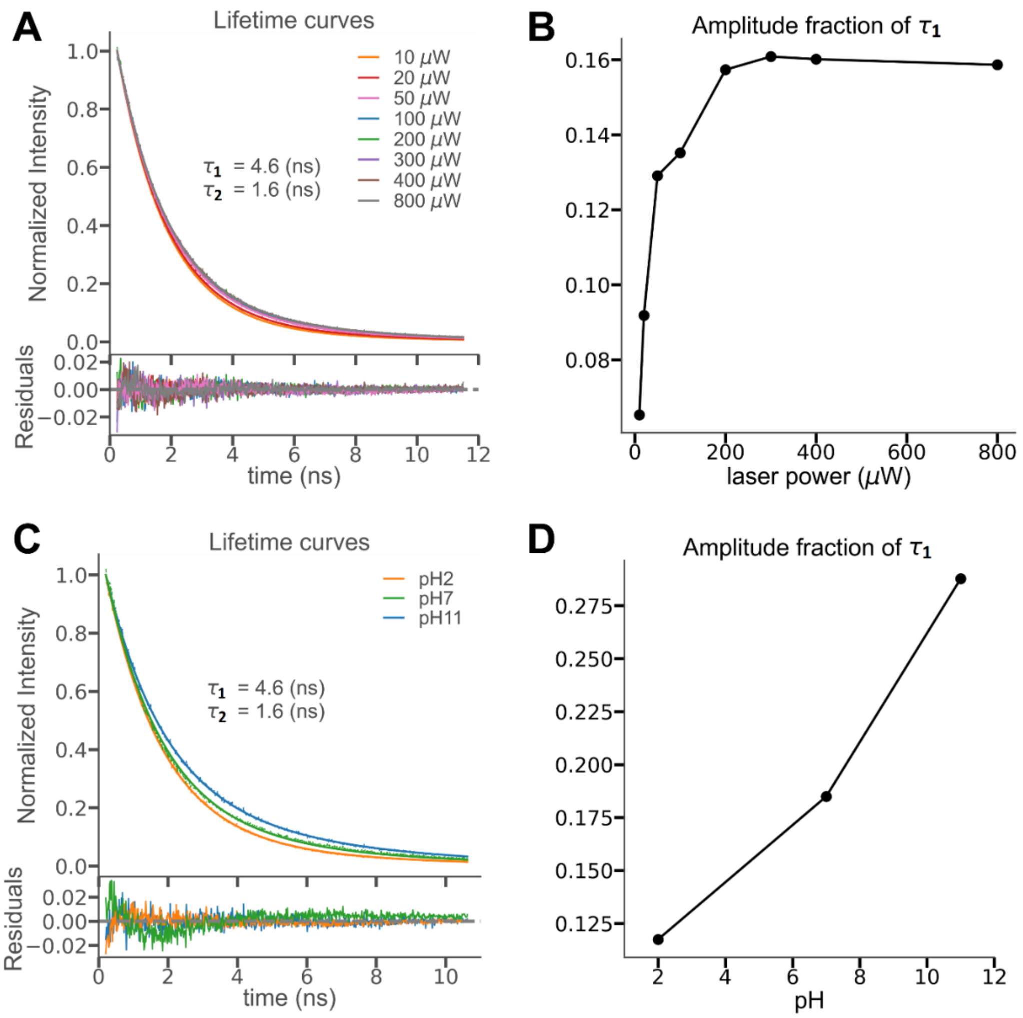

Figure S2. *Time-correlated single photon counting (TCSPC) fluorescence lifetime measurements recorded from a DPBS buffer (Sigma-Aldrich, cat. no. D8537, air atmosphere) with POPC small unilamellar vesicles (SUVs) labeled with 500nM NAO. (A) Fluorescence decay measurements by TCSPC (pH 7.4) with different excitation laser powers applied. The decay curves were fitted as bi-exponential decays, from which two lifetimes (τ_1 = 4.6 ns and τ_2 = 1.6 ns, fitted globally) and their relative amplitude fractions could be extracted. The relative amplitude fractions of τ_1 (B) increased with I_{exc}. (C) TCSPC curves (200 μW laser excitation) recorded from NAO labled SUVs (as in (A)), in DPBS buffer with different pH. The curves were fitted as bi-exponential decays using the two lifetimes from (A) (τ_1 = 4.6 ns and τ_2 = 1.6 ns), from which their relative amplitude fractions could be extracted. The relative amplitude fraction of τ_1 (D) increased with pH. In the TCSPC measurements, each SUV contained multiple NAO fluorophores, so that the SUVs can be considered to have isotropic excitation and emission dipole moments. Moreover, no polarizers were used in front of the detectors in the measurements. Therefore, any anisotropy decay effects on the observed fluorescence decays should be minor and could be disregarded.*

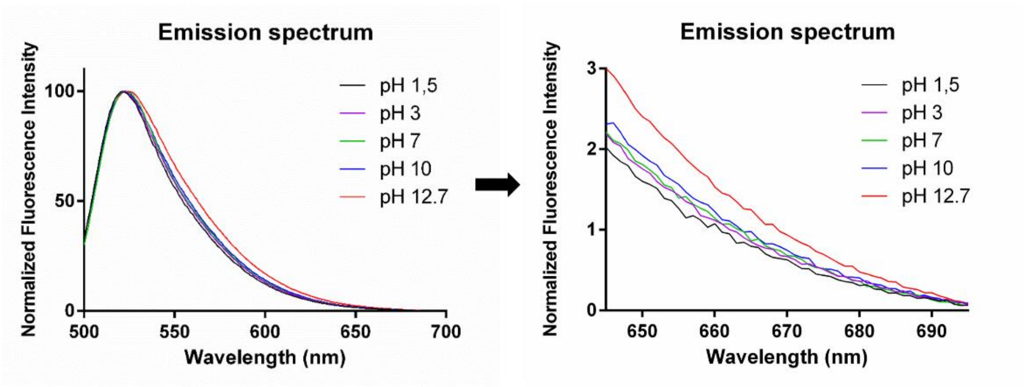

Figure S3. *Emission spectrum recorded from POPC small unilamellar vesicles (SUVs) labeled with 250nM NAO in DPBS buffer (Sigma-Aldrich, cat. no. D8537, air atmosphere) under different pH. The emission spectrum from 500nm to 700nm (left). Zoom-in of the emission spectrum in the range 650nm to 700nm (right).*

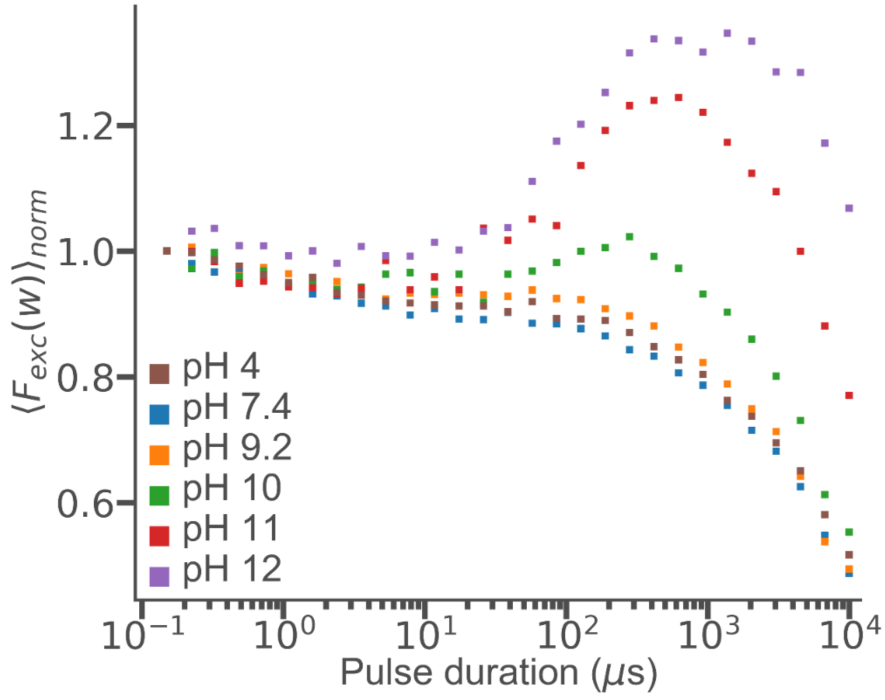

Figure S4. *Experimental TRAST curves recorded from 1 μM AO in DPBS buffer (Sigma-Aldrich, cat. no. D8537, air atmosphere) with different pH.*

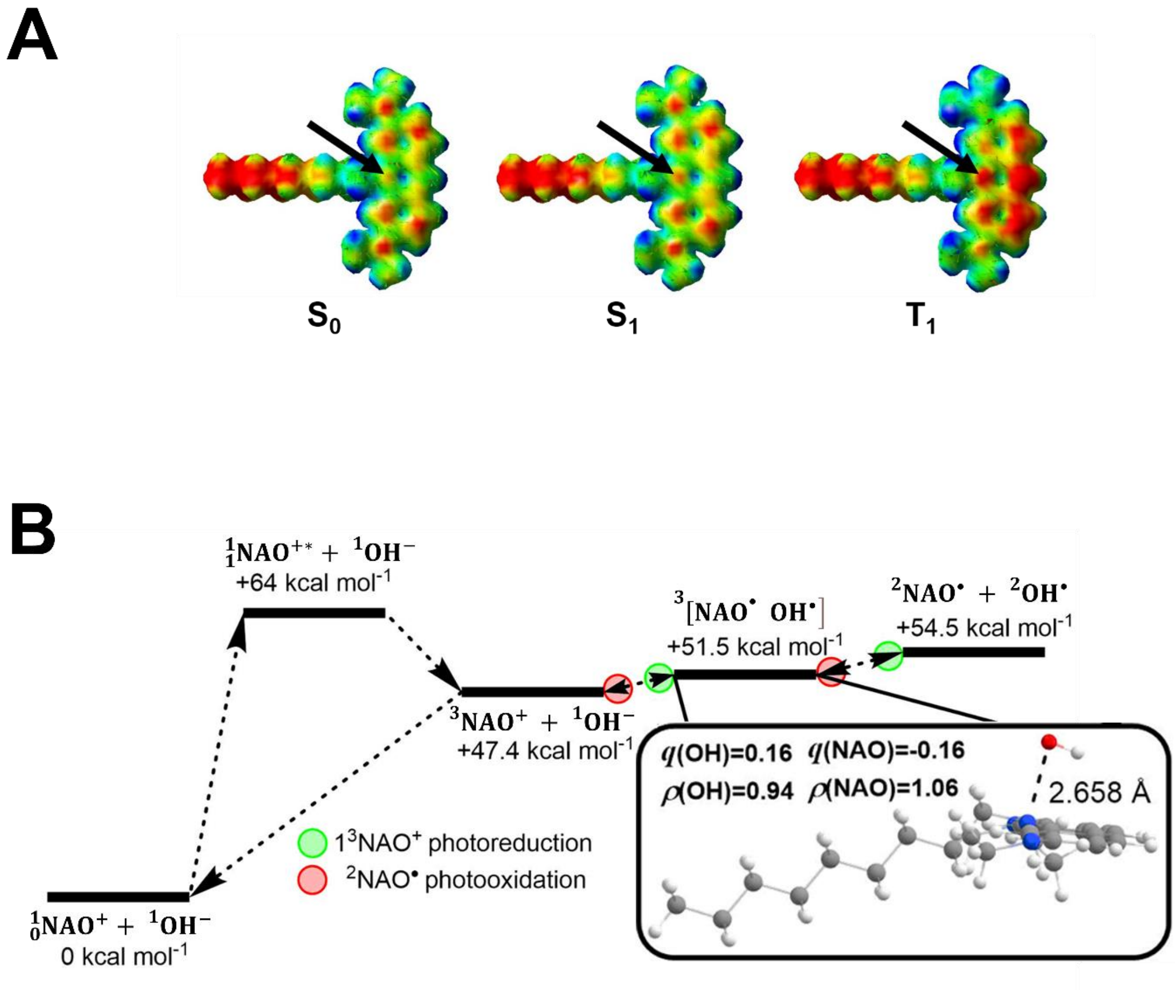

Figure S5. *(A) Molecular electrostatic potential (MESP) plots for the NAO^+ dye in the ground (S_0) and excited singlet (S_1) and triplet (T_1) states. Red colour denotes more positive ESP. The T_1 state NAO^+ sustains a more positive ESP over the intracyclic nitrogen atom. When NAO^+ is in the T_1 state it is thus a stronger Lewis acid, compared to when it is in the S_0 or S_1 state. (B) Energy diagram for the formation of the triplet state $^3[NAO^\bullet\ OH^\bullet]$ intermediate, which upon dissociation leads to the formation of the red-emissive $^2NAO^\bullet$ species. q(x) refers to the Mulliken atomic charge of species x and ρ(x) to the corresponding Mulliken spin density. The energy values in kcal mol^{-1} refer to the relative relaxed energies of key intermediates between the initial reagents (0 kcal mol^{-1}) and products (+51.5 kcal mol^{-1}).*

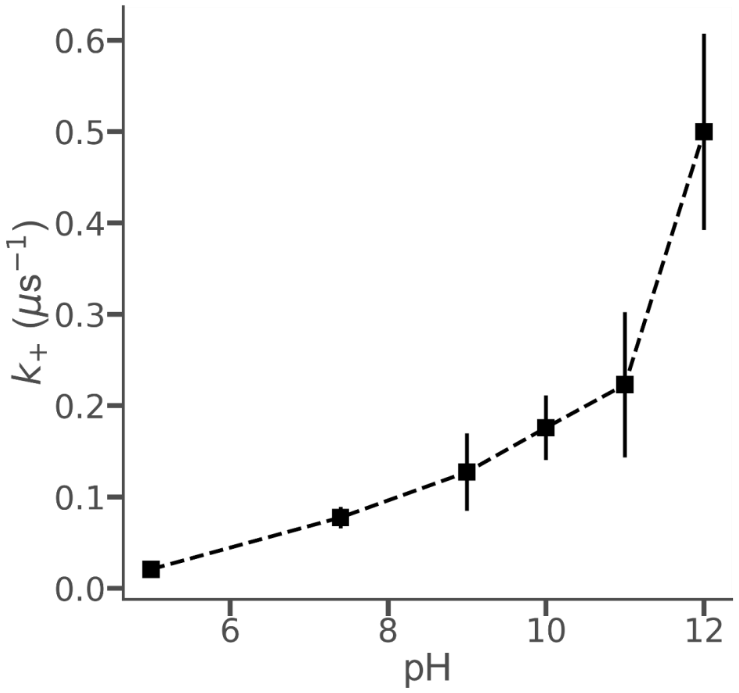

Figure S6. *The fitted k_+ rates from the TRAST measurements in Figure 2D.*

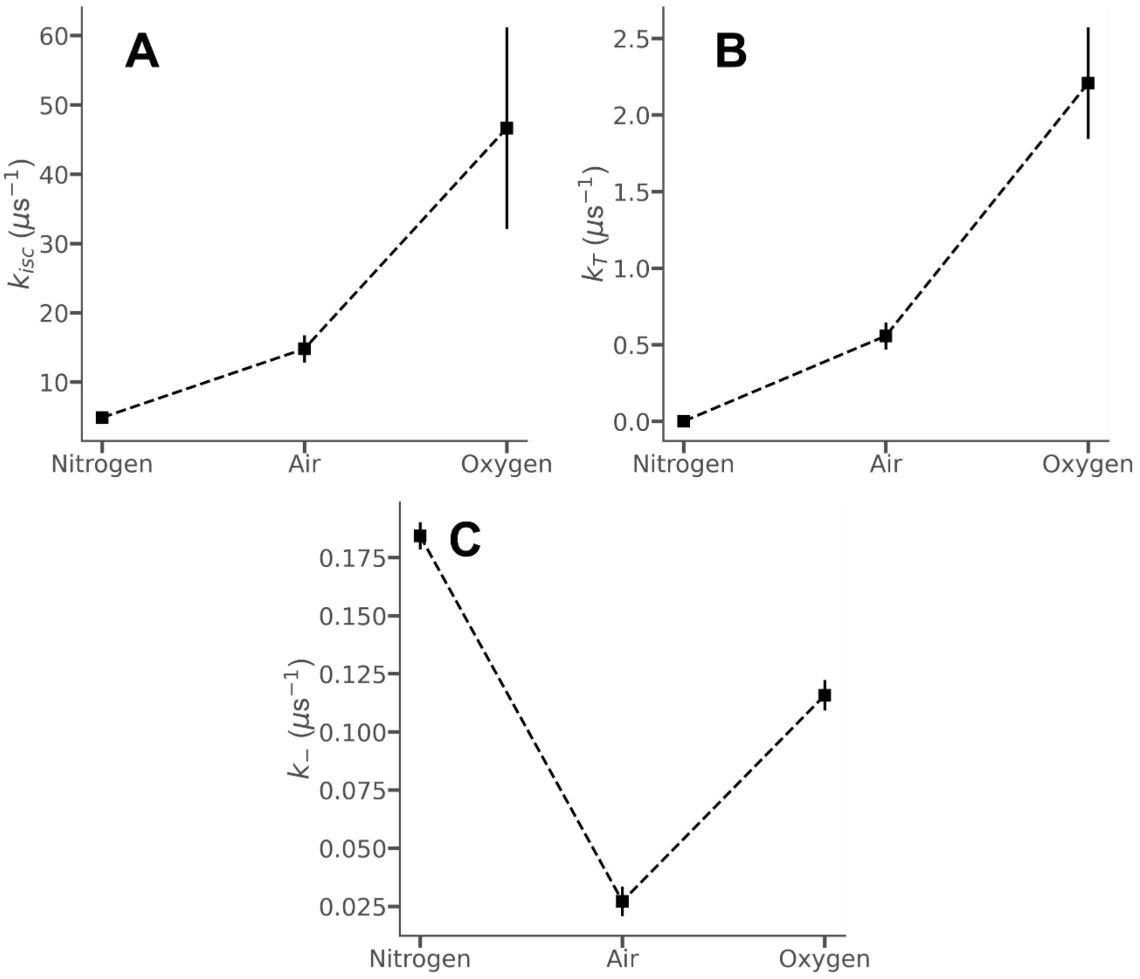

Figure S7. *The fitted rates k_{isc}, k_T and k_- from TRAST measurements in Figure 2B are shown in (A), (B) and (C) respectely.*

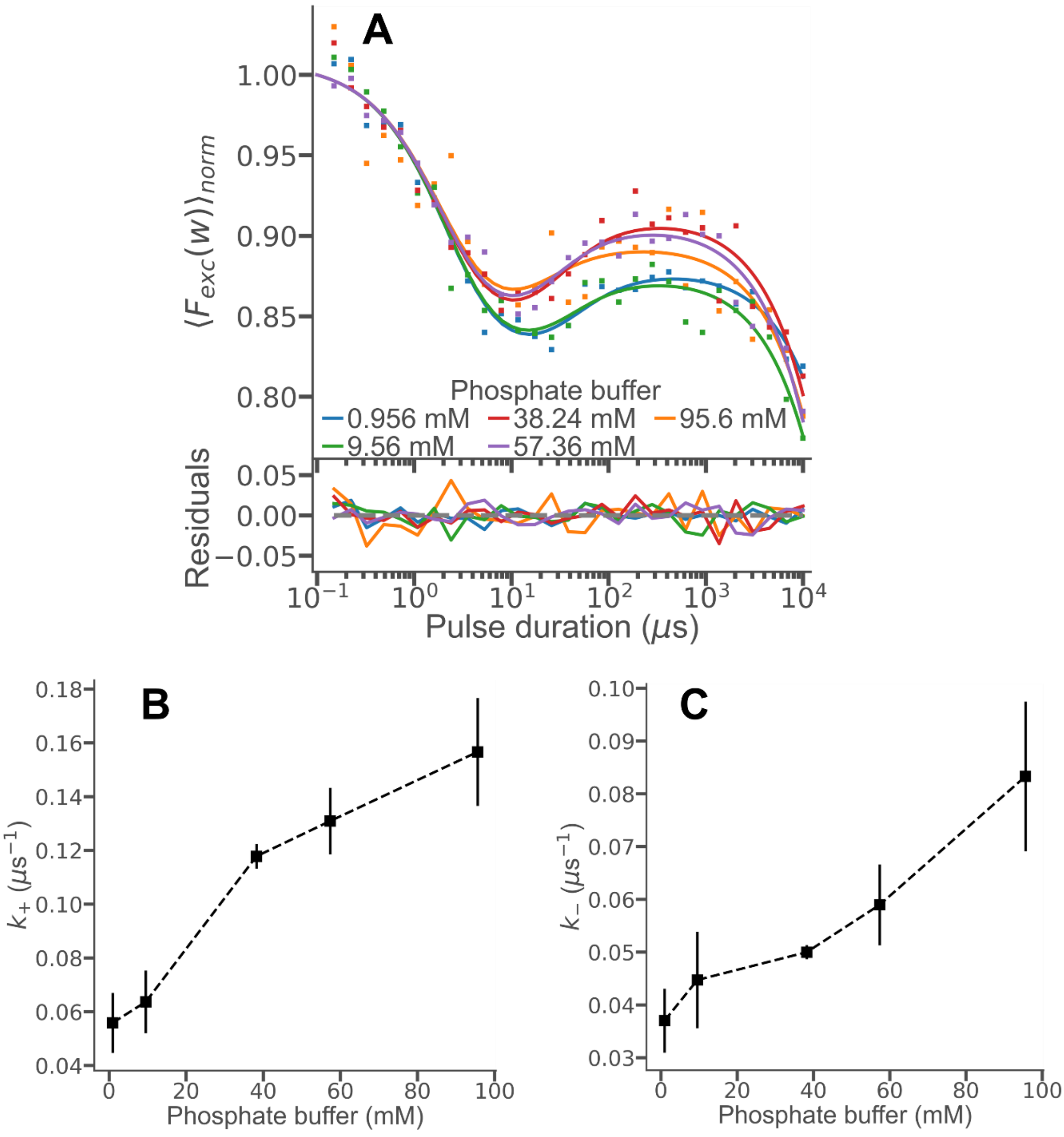

Figure S8. *TRAST curves (A) of NAO in POPC vesicles with varying* concentrations of a phosphate buffer *(air atmosphere, pH 7.4, measured at 11.7kW/cm*^{2}*). TRAST curves are fitted globally using the photophysical model (Figure 3B). The fitted rates* k_+ *and* k_- *from TRAST measurements are showed in (B) and (C) respectely.*

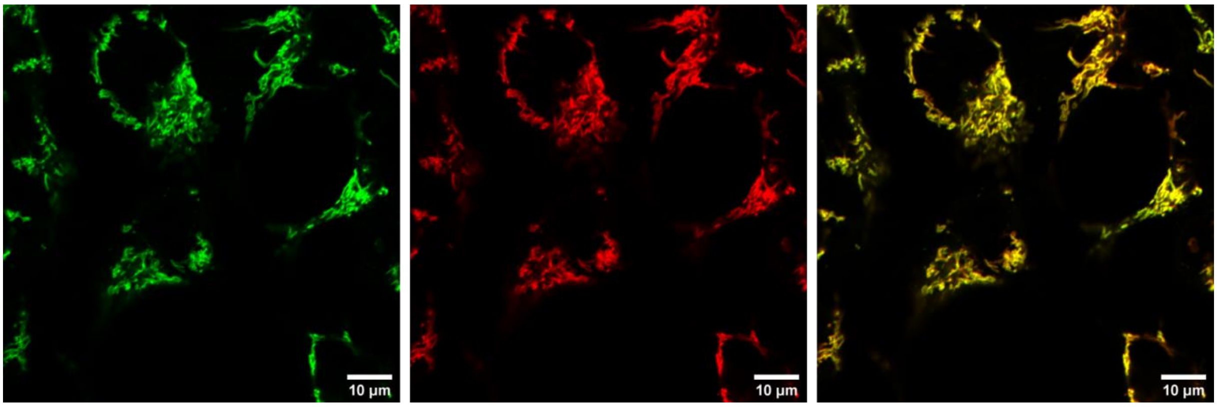

Figure S9. *Confocal image of Hek293A cells labeled with 250nM NAO, detected in the green channel (left, 500nm to 570nm) and 200nM Mitotracker Red, detected in the red channel (middle, 645nm to 695nm). Right: overlaid image, showing high degree of co-localization of the two fluorophores, in the mitochondrial membranes of the cells.*

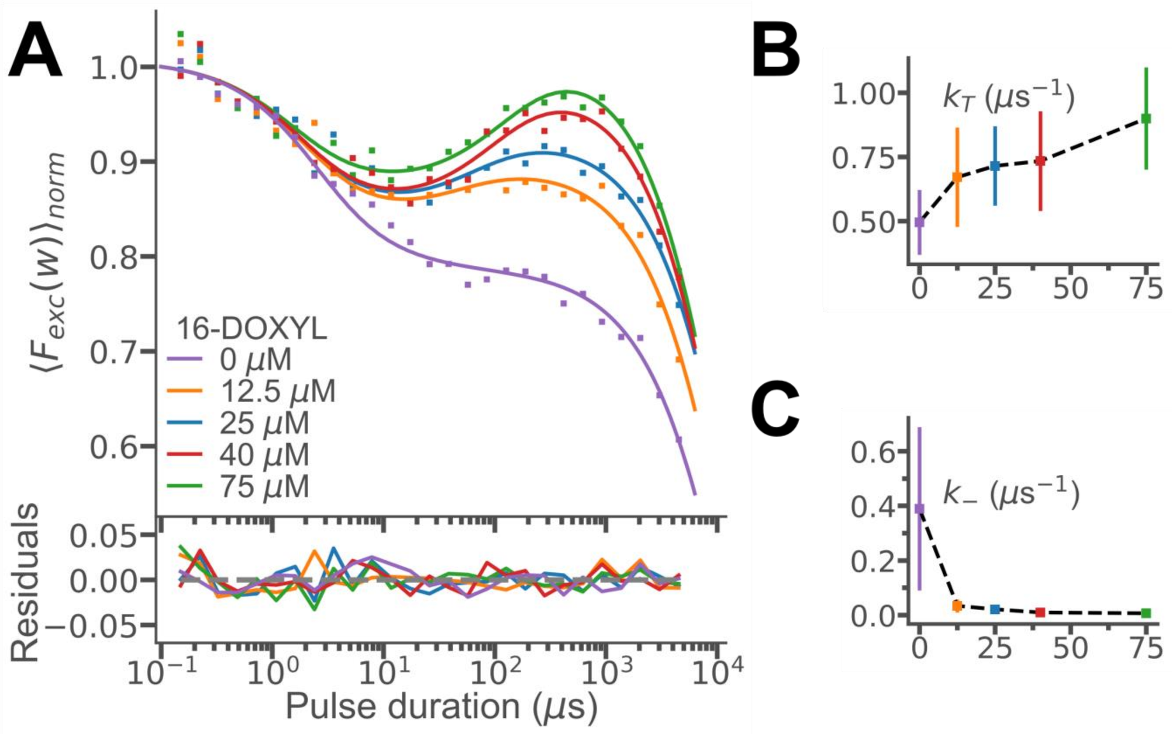

Figure S10. *TRAST curves generated from the total fluorescence intensity in images recorded from HEK293A cells. These curves were very similar to the curves recorded from POPC vesicles with 15% (18:2)$_4$CL (Figure 5B). (A) With 16-Doxyl added into the cells, a significant decrease in the initial decay and an increase in the negative relaxation amplitude in these TRAST curves can be observed. The curves were globally fitted, in the same way as the curves from the vesicle sample, with 16-Doxyl added (Figure S11A). k_+ was globally fitted (0,053μs^{-1}), while k_T and k_- were fitted individually to each curve. In agreement with the vesicle measurements, we found that k_T (B) increases and k_- (C) decreases with increasing 16-Doxyl concentrations added to the cells.*

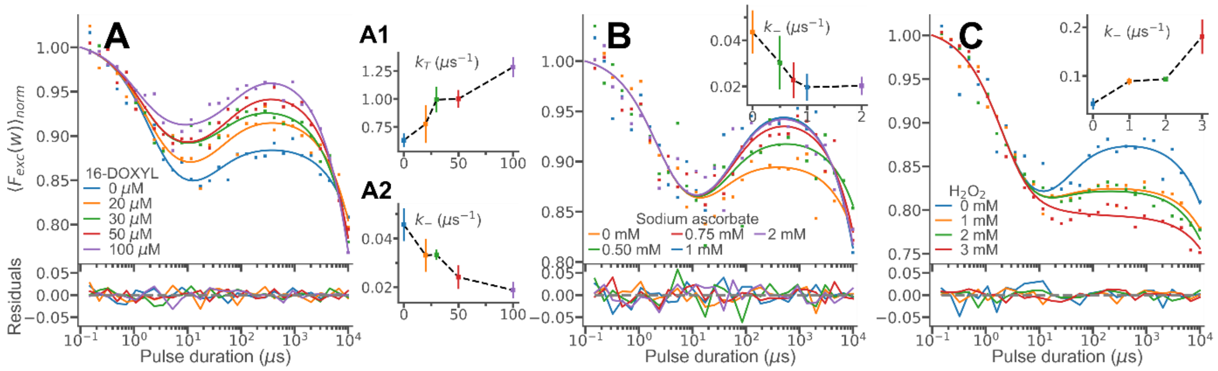

Figure S11. *Experimental TRAST curves recorded from POPC SUVs labeled with NAO in DPBS buffer (pH 7.4, air atmosphere, and at I_{exc} 11.7kW/cm^2). TRAST curves were fitted globally using the photophysical model (Figure 3B), see main text for details. (A) TRAST curves recorded upon adding a spin-label quencher (16-doxyl stearic acid), together with fitted curves, and with the individually fitted rates k_T and k_- shown in the insets A1 and A2, respectively. (B) Recorded TRAST curves upon addition of sodium ascorbate, with the individually fitted k_ rates shown in the inset. (C) TRAST curves recorded from samples with H_2O_2 added. The individually fitted k_ rates are shown in the inset.*

	**Rate** μs^{-1}
Q	0.40 ± 0.011
k_{isc}	16.2 ± 2.8
k_T	0.58 ± 0.16
k_+	0.077 ± 0.012 (at pH7.4)
k_-	0.042 ± 0.0079

Table S1. *Photophysical rate parameter values determined by global fitting of TRAST curves from Figure 2D. These values were used to fix parameters in the subsequent global fitting of recorded TRAST curves, as described in the main text. The uncertainties are estimates and represent the standard error of the mean for different measurements when the other parameters in the table are fixed in the fitting.*

References

(1) Tornmalm, J.; Sandberg, E.; Rabasovic, M.; Widengren, J. Local redox conditions in cells imaged via non-fluorescent transient states of nad(p)h. *Sci. Rep.* **2019,** *9,* 15070.
(2) Wiosetek-Reske, A. M.; Wysocki, S. Spectral studies of n-nonyl acridine orange in anionic, cationic and neutral surfactants. *Spectrochim. Acta. A Mol. Biomol. Spectrosc.* **2006,** *64,* 1118-1124.
(3) Zhao, Y.; Truhlar, D. G. The m06 suite of density functionals for main group thermochemistry, thermochemical kinetics, noncovalent interactions, excited states, and transitions elements: Two new functionals and systematic testing of four m06-class functionals and 12 other functionals. *Theor. Chem. Acc.* **2008,** *120,* 215-241.
(4) Ditchfield, R.; Hehre, W. J.; Pople, J. A. Self-consistent molecular-orbital methods. Ix. An extended gaussian-type basis for molecular-orbital studeis of organic molecules. *J. Chem. Phys.* **1971,** *54,* 724-728.
(5) Clark, T.; Chandrasekhar, J.; Spitznagel, G. W.; Schleyer, P. V. Efficient diffuse function-augmented basis sets for anion calculations. Iii. The 3-21+g basis set for first-row elements, li–f. *J. Comput. Chem.* **1983,** *4,* 294-301.
(6) Frisch, M. J.; Pople, J. A.; Binkley, J. S. Self - consistent molecular orbital methods 25. Supplementary functions for gaussian basis sets. *J. Chem. Phys.* **1984,** *80,* 3265-3269.
(7) Tomasi, J.; Mennucci, B.; Cammi, R. Quantum mechanical continuum solvation models. *Chem. Rev.* **2005,** *105,* 2999-3093.
(8) Marenich, A. V.; Cramer, C. J.; Truhlar, D. G. Universal solvation model based on solute electron density and on a continuum model of the solvent defined by the bulk dielectric constant and atomic surface tensions. *J. Phys. Chem. B* **2009,** *113,* 6378-6396.
(9) Thapa, B.; Schlegel, H. B. Density functional theory calculation of pk(a)'s of thiols in aqueous solution using explicit water molecules and the polarizable continuum model. *J. Phys. Chem. A* **2016,** *120,* 5726-5735.
(10) Sutton, C. C. R.; Franks, G. V.; da Silva, G. First principles pk(a) calculations on carboxylic acids using the smd solvation model: Effect of thermodynamic cycle, model chemistry, and explicit solvent molecules. *J. Phys. Chem. B* **2012,** *116,* 11999-12006.
(11) Adamo, C.; Jacquemin, D. The calculations of excited-state properties with time-dependent density functional theory. *Chem. Soc. Rev.* **2013,** *42,* 845-856.
(12) Kellmann, A.; Lion, Y. Acid-base equilibria of the excited singlet and triplet-states and the semi-reduced form of acridine-orange. *Photochem. Photobiol.* **1979,** *29,* 217-222.
(13) Dorfman, L. M.; Adams, G. E. Reactivity of the hydroxyl radical in aqueous solutions. **1973**, *46,* 1-59.
(14) Valiev, R. R.; Cherepanov, V. N.; Baryshnikov, G. V.; Sundholm, D. First-principles method for calculating the rate constants of internal-conversion and intersystem-crossing transitions. *Phys. Chem. Chem. Phys.* **2018,** *20,* 6121-6133.
(15) Widengren, J.; Mets, U.; Rigler, R. Fluorescence correlation spectroscopy of triplet states in solution: A theoretical and experimental study. *J. Phys. Chem.* **1995,** *99,* 13368-13379.
(16) Strömqvist, J.; Chmyrov, A.; Johansson, S.; Andersson, A.; Mäler, L.; Widengren, J. Quenching of triplet state fluorophores for studying diffusion-mediated reactions in lipid membranes. *Biophys. J.* **2010,** *99,* 3821-3830.
(17) Widengren, J.; Chmyrov, A.; Eggeling, C.; Löfdahl, P. Å.; Seidel, C. A. M. Strategies to improve photostabilities in ultrasensitive fluorescence spectroscopy. *J. Phys. Chem. A* **2007,** *111,* 429-440.
